# Fiberbots: Robotic fibers for high-precision minimally invasive surgery

**DOI:** 10.1126/sciadv.adj1984

**Published:** 2024-01-19

**Authors:** Mohamed E. M. K. Abdelaziz, Jinshi Zhao, Bruno Gil Rosa, Hyun-Taek Lee, Daniel Simon, Khushi Vyas, Bing Li, Hanifa Koguna, Yue Li, Ali Anil Demircali, Huseyin Uvet, Gulsum Gencoglan, Arzu Akcay, Mohamed Elriedy, James Kinross, Ranan Dasgupta, Zoltan Takats, Eric Yeatman, Guang-Zhong Yang, Burak Temelkuran

**Affiliations:** ^1^ The Hamlyn Centre for Robotic Surgery, Imperial College London, London SW7 2AZ, UK.; ^2^ Department of Electrical and Electronic Engineering, Faculty of Engineering, Imperial College London, London SW7 2AZ, UK.; ^3^ Department of Metabolism, Digestion and Reproduction, Faculty of Medicine, Imperial College London, London SW7 2AZ, UK.; ^4^ Department of Mechanical Engineering, Inha University, Incheon 22212, South Korea.; ^5^ The Rosalind Franklin Institute, Didcot OX11 0QS, UK.; ^6^ The UK DRI Care Research and Technology Centre, Department of Brain Science, Imperial College London, London W12 0MN, UK.; ^7^ Institute for Materials Discovery, University College London, London WC1H 0AJ, UK.; ^8^ Department of Mechatronics Engineering, Faculty of Engineering, Yildiz Technical University, Istanbul 34349, Turkey.; ^9^ Department of Dermatology and Venereology, Liv Hospital Vadistanbul, Istanbul 34396, Turkey.; ^10^ Department of Skin and Venereal Diseases, Faculty of Medicine, Istinye University, Istanbul 34010, Turkey.; ^11^ Department of Pathology, Faculty of Medicine, Yeni Yüzyıl University, Istanbul 34010, TR.; ^12^ Pathology Laboratory, Atakent Hospital, Acibadem Mehmet Ali Aydinlar University, Istanbul 34303, TR.; ^13^ Anesthesiology, University Hospitals of Derby and Burton, Derby, DE22 3NE, UK.; ^14^ Department of Surgery and Cancer, Faculty of Medicine, Imperial College London, London SW7 2AZ, UK.; ^15^ Department of Urology, Imperial College Healthcare NHS Trust, Charing Cross Hospital, London W6 8RF, UK.; ^16^ Institute of Medical Robots, Shanghai Jiao Tong University, Shanghai 200240, China.

## Abstract

Precise manipulation of flexible surgical tools is crucial in minimally invasive surgical procedures, necessitating a miniature and flexible robotic probe that can precisely direct the surgical instruments. In this work, we developed a polymer-based robotic fiber with a thermal actuation mechanism by local heating along the sides of a single fiber. The fiber robot was fabricated by highly scalable fiber drawing technology using common low-cost materials. This low-profile (below 2 millimeters in diameter) robotic fiber exhibits remarkable motion precision (below 50 micrometers) and repeatability. We developed control algorithms coupling the robot with endoscopic instruments, demonstrating high-resolution in situ molecular and morphological tissue mapping. We assess its practicality and safety during in vivo laparoscopic surgery on a porcine model. High-precision motion of the fiber robot delivered endoscopically facilitates the effective use of cellular-level intraoperative tissue identification and ablation technologies, potentially enabling precise removal of cancer in challenging surgical sites.

## Teaser

Heat-controlled fiber robots could potentially improve the precision of minimally invasive cancer surgery.

## INTRODUCTION

The main goal of surgical oncology is to achieve complete resection of cancerous tissue with minimal iatrogenic injury to surrounding healthy tissue. This presents a formidable challenge to surgeons, especially in minimally invasive surgery (MIS). While removing a tumor, the surgeon needs to resect it with a margin to include adjacent healthy tissue for histopathological assessment. Exemplar cases include hepatic resections due to colorectal metastasis, where the primary objective involves excising cancerous liver tissue with recommended large optimal margins (resulting in removal of >1-cm healthy tissue surrounding the tumor) to minimize the risk of cancer recurrence post-operatively (*1*, *2*)*.* Balancing the resection position is crucial, as excessive liver tissue removal can lead to postoperative complications or even liver failure (*3*)*.* The tumor’s proximity to critical structures such as the bile duct will additionally complicate achieving wide margins and safe removal of the tumor (*4*)*.*

**Fig. 1.**
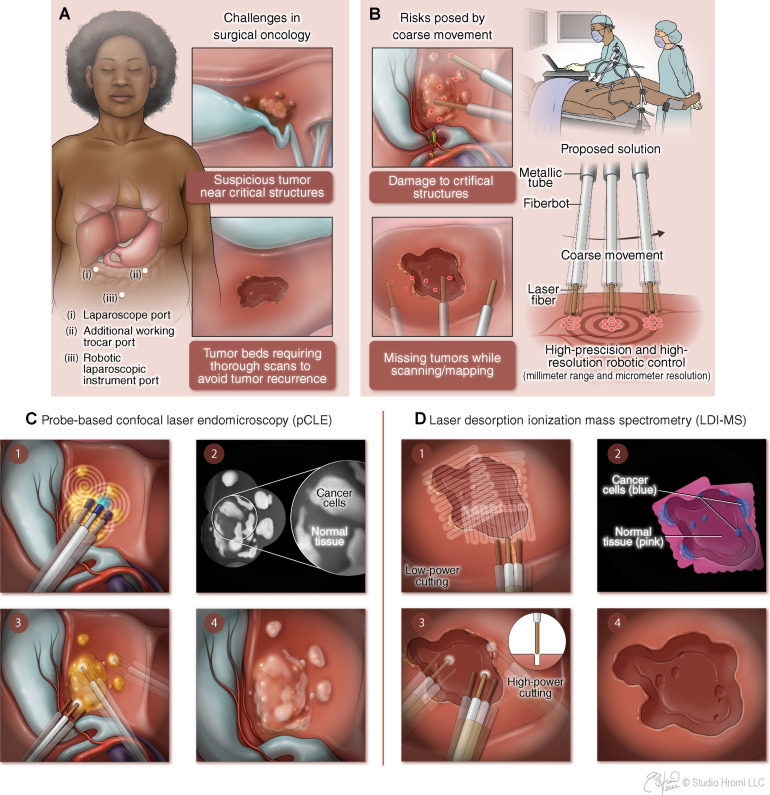
Clinical challenges in laparoscopic surgery and our proposed solution. (**A**) Abdominal laparoscopic surgery and the two major challenges: a tumor within the vicinity of critical structures and missed remnants of diseased tissue following tumor removal. (**B**) Fiberbot is introduced through the laparoscopic instrument, stabilized by a simple clamp mechanism, introducing computer-controlled precise motion. (**C**) Example of how the fiberbot can mitigate the risks of damage to nearby structures by maneuvering a pCLE probe while mapping the surgical site. Following fluorescent dye injection via the fiberbot’s working channel (1), a pCLE probe is advanced through the same channel, and the target area is scanned (2). The laparoscopic instrument then directs the fiberbot to scan multiple locations covering the target area (3), providing the surgeon with a microscopic histopathological view of the site that guides surgical decision-making (4). (**D**) Fiberbot is used with an laser desorption–rapid evaporative ionization mass spectroscopy (LD-REIMS) system to reveal and remove the possible missed remnants of diseased tissue following tumor removal. A laser fiber is advanced through the working channel of the fiberbot, and a suction tube is attached to it, directing the surgical aerosol to for mass spectrometry identification (1). A high-resolution molecular map of the region of interest with detailed tissue information is presented to the surgeon (2). Fiberbot targets and ablates (autonomous or tele-operated) any remnants of diseased tissue located on the map (3 and 4).

To resolve these challenges, researchers have been focusing on introducing high-precision surgical instruments such as: (i) surgical tools for more precise tissue dissection, (ii) cellular-level intraoperative diagnostic devices, and (iii) robotic instruments. For soft tissue resection, infrared surgical lasers such as carbon dioxide (CO_2_) lasers flexibly delivered by special hollow core fibers (*5*, *6*), enhance MIS by providing superior precision in tissue dissection with minimized thermal spread to adjacent healthy tissues (<100 μm) when compared to standard surgical dissection tools such as diathermy (*7*)*.* For intraoperative sensing, emerging cellular-level diagnostic technologies have great potential for intraoperative tissue identification and characterization at the microscopic level (*8**–**11*). Integration of therapeutic and diagnostic tools in MIS with high-precision robotic instruments that can maneuver these tools during surgical interventions is quite evident to allow surgeons to operate more confidently around critical structures while minimizing the possibility of leaving diseased tissue remnants at the surgical site and reducing the amount of healthy tissue currently excised for manual margin assessment (Fig. 1A).

Several MIS robotic platforms have been proposed recently with different actuation mechanisms that can potentially address the challenges faced by surgical oncology, among other clinical interventions (*12*)*.* Hence, actuation with tendon-driven steerable devices provides excellent tip coverage and workspace. One typical example is the Da Vinci SP (Intuitive Surgical Inc., USA) system that has been widely applied in various MIS such as head-and-neck and gynecological surgeries (*13*) as a standalone operating platform or integrated with diagnostic sensing systems for in vivo tissue assessment (*14*)*.* However, as highlighted by Lee *et al.* (*15*), nonlinear friction issues, including backlash hysteresis, dead zones from wire slack, and plastic torsion, still constrain the performance of tendon-driven instruments. The robot proposed by Kundrat *et al.* (*16*), with a motion range of 45 mm by 45 mm, is limited by its positional errors in the hundreds of micrometers, resulting from the aforementioned constraints. The work by Zhao *et al.* (*17*), although offering higher precision [root mean square (RMS) error of 0.054 ± 0.028 mm] within a 28ϕ-mm motion range, is constrained by their larger radial size of 22 mm in diameter, affecting their application in compact environments*.*

Magnetically actuated probes have proven their efficacy in various surgical fields that require robotic transfer through complex pathways, including fetal (*18*), lung (*19*), eye (*20*), and endovascular surgeries (*21**–**23*). A variant magnetic probe, with a 13 mm in diameter, achieves rapid response (maximum speed of 94 mm/s) and a trajectory tracing accuracy of 0.09 mm. However, because of the size of the coils and permanent magnet within the device, its range of motion is restricted to just 4 mm by 4 mm (*24*)*.* In contrast, the smaller 0.4-mm-diameter magnetic actuator developed by Charreyron *et al.* (*25*) is advantageous in applications requiring minimal size, such as their proposed eye surgery. Using a closed-loop navigation approach, this magnetic actuator enables accurate motion with estimated average errors ranging in the hundreds of micrometers. However, their magnetic systems require either large electromagnets [OctoMag used in this example (*25*)] or permanent magnets (such as commercialized Stereotaxis Genesis RMN system) for actuation. Consequently, this can pose magnetic interference issues in environments sensitive to magnetic fields while requiring large dedicated operating theaters.

Other comparable robotic devices are hydraulically actuated microactuators, microrobotic tentacles, and microcatheters (*26**–**28*)*.* In general, soft robots driven by hydraulics have great dexterity, have high-power density, and can exert large application forces. Hydraulic actuator developed by Gopesh *et al.* (*28*) with a diameter of 0.9 mm, offers a combination of safety and ability in traversing tortuous routes but faces an estimated RMS error in the hundreds of micrometers. Their functionality is complicated by the nonlinear behavior of soft materials, adding complexity to their application in precision-critical environments.

Concentric tube robots (CTRs), with the manipulation of the precurved nested tubes (for rotation and translation), enable bending and twisting movements, resulting in miniature and flexible platforms. CTRs have been demonstrated in several challenging surgical contexts, including the brain (*29*), larynx (*30*), and liver (*31*) surgeries. The robot Caturo developed by Nwafor *et al.* (*32*) represents one of the most recent advancements in CTR. It is compatible with various inner tube deployment methods, such as spiral and helical curvatures. The Caturo robots exhibit a range of curvature radii from 5 mm to 63 mm and have an outer diameter (OD) of less than 200 μm for their inner tubes, enabling a positional RMS error of 0.201 mm. However, as concluded by Alfalahi *et al.* (*33*), concerns persist regarding potential bifurcation risks, the internal friction, shear, and axial elongation in these robotic systems. The CTR systems also need an actuation unit (such as a lead screw linear actuator) that is directly connected to the tubes to generate their axial motion.

By its turn, the mirror steering robots control the position of a laser spot by adjusting mirrors or prism angles. This approach can achieve laser scanning speeds exceeding 100 mm/s (*34*, *35*), with the micro-robotic laser steering device developed by York *et al*. (*36*) achieving a remarkable speed of 3900 mm/s*.* Nonetheless, these devices require complex assembly of the different miniaturized parts composing the robot itself, while the achievable precision is primarily confined to the millimeter level. It is also challenging to avoid laser-induced damage to the mirrors due to contamination of their surfaces.

The use of nonstandard and electrically active materials is yet another approach, encompassing piezoelectric materials and electroactive polymers. Polymer-based electrostrictive actuators proposed by Khudiyev *et al.* (*37*) have dimensions ranging from 0.24 to 0.46 mm in thickness and 0.68 to 1.3 mm in width*.* Although these actuators provide submicrometer precision, they are constrained by a narrow motion range of 0.08 mm and the requirement for high actuation voltage*.* These constraints limit their application mainly to scenarios that require highly precise, small-scale movements. While piezoelectric actuation mechanisms have higher precision at micrometer levels (*38*), they require high voltage levels and long rigid materials that affect flexible access to body cavities and passages.

Actuators that use thermal expansion mechanisms have recently demonstrated substantial potential in achieving precise displacements in several applications. These actuators typically use the energy generated from an energized resistance or light beam as a heat source to control the temperature and the volume of a single or multiple materials to achieve actuation. These actuation mechanisms have been deployed to perform tasks that require high-precision operation with delicate structures, such as optical fiber cantilevers and microelectromechanical systems for micro- and nanomanipulation (*39**–**41*)*.* More recently, thermal actuators have also demonstrated flexibility in soft actuation devices for untethered robots (*42*, *43*) and artificial muscle control (*44**–**46*)*.* However, the precise displacement potential of thermal actuation has not yet been harnessed to address the unmet needs in MIS (*47*)*.*

In this work, we present an electrothermally actuated polymer-based fiber device, which is a long (~10 cm) and thin (<2 mm in diameter) robot that enables high-precision motion control for MIS (30-μm step motion, with average path error below 50 μm, speeds of 10 mm/s). We will refer to this device as the “fiberbot” because it is fabricated using the thermal fiber drawing process, which is becoming an emerging technology in the scalable fabrication of structurally flexible and intrinsically integrable medical devices (*48*, *49*)*.* In a clinical scenario, this fiberbot can be deployed to the surgical site via standard interventional instruments such as laparoscopic devices, enabling coarse positioning of the fiberbot within the proximity of the target tumor area (Fig. 1B). Equipped with endomicroscopy fiber bundle probes placed inside the working channel, it enables accurate subcellular identification of suspicious cancerous areas (Fig. 1C). By its turn, the fiberbot integrated with a surgical CO_2_ laser and intraoperative tissue identification system using mass spectrometric analysis of generated surgical aerosol (Fig. 1D) enables precise localization and ablation of the target tissue with efficient removal of possible tumor remnants at the margins.

When compared to other devices, the fiberbot (i) can achieve 30-μm resolution over several millimeters of motion range with a high-aspect ratio of robot body (>50:1); (ii) with a simple design and material choices (i.e., common thermoplastics and metal wires), allows mass production (hundreds of meters of fiberbots per fiber draw); (iii) does not require a large actuation unit for manipulation; (iv) does not have any mechanical components; and (v) presents negligible material hysteresis. Furthermore, tables S1 and S2 from the Supplementary Materials provide additional comparison metrics for motion performance (range and precision) and electronic activation between the proposed fiberbot and other state-of-the-art robotic alternatives published in literature for clinical applications.

**Fig. 2.**
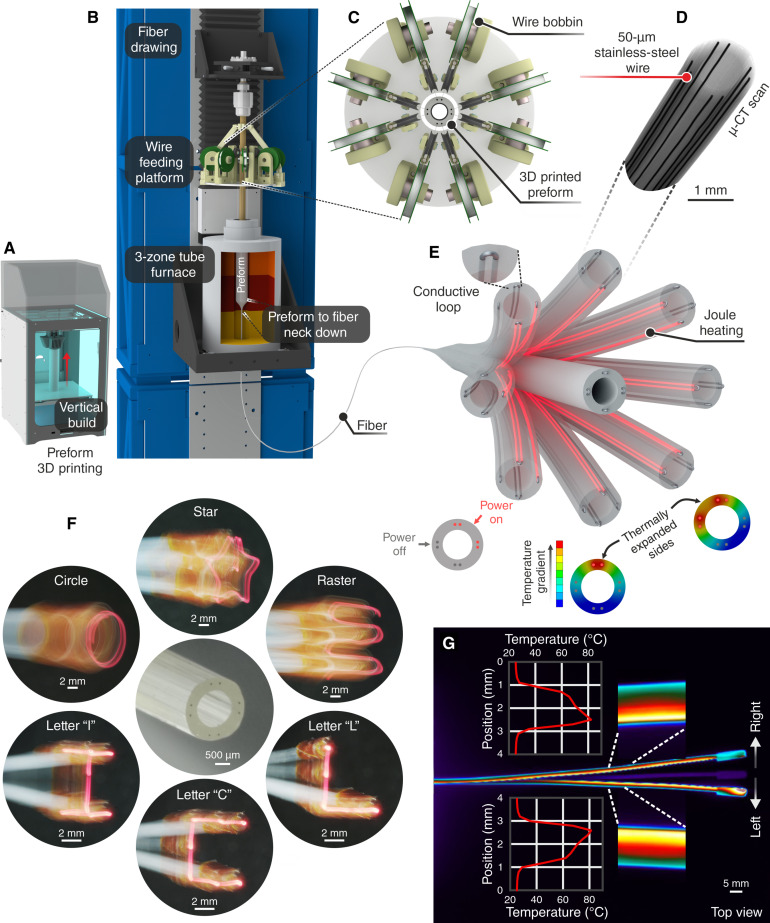
Fabrication and working principle of the fiberbot. (**A**) Preform fabrication process via 3D printing. (**B**) Electrothermally actuated fibers are produced by thermal fiber drawing. (**C**) Top view of the wire feeding platform used to guide eight stainless-steel wires into the preform’s side channels. (**D**) Micro-computed tomography (μ-CT) scan of the thermally drawn fiber. (**E**) Illustration of the fiber’s cantilever bending, which is powered by thermal expansion. Inset: Exposed wires at the fiber’s distal end intertwined in pairs and coated with silver paint to form a conductive loop. (**F**) Six tip displacement patterns captured using a mirrorless camera’s slow shutter speed function while moving a 500-μm optical fiber connected to a 650-nm light-emitting diode (LED). (**G**) Temperature distribution profile along the fiber’s length obtained by a thermal camera during bending movements.

This work starts by laying down the soft actuator’s design, followed by the fabrication process via thermal drawing technology (Fig. 2, A to C). The resulting fiberbot encompasses a thin-walled polymeric fiber embedded with thin metallic resistive wires (Fig. 2D) capable of generating a thermal gradient across the cross section of the fiber (Joule heating), thereby inducing an asymmetric expansion of the fiber and the desired motion of its tip (Fig. 2E). Second, we present a rapid prototyping approach that simplifies the production of hundreds of meters of these inexpensive, bespoke, and integrable soft actuators that can readily be operated. Next, to control these actuators, we implement open-loop and closed-loop controllers to trace predefined trajectories accurately (Fig. 2F). Then, the benefits of the fiberbot’s precise motion capacity are made evident by combining with emerging intraoperative diagnostic technologies providing information at the microscopic level. By performing highly precise scans using the fiberbot, we were able to generate high-resolution molecular distribution maps of mouse brain tissue and large, microscopic field-of-view (FOV) areas of bladder cancer tissue. At the end, we successfully deployed a longer fiberbot into the abdomen of a porcine model during in vivo surgery through an imitated laparoscopic instrument. In this setting, we demonstrated that the precise motion can be easily and safely delivered to achieve precise tissue ablation in an MIS setting, further reinforcing the potential of the proposed fiberbot for clinical utility.

## RESULTS

### Design and scalable fabrication of the high-precision fiberbots

The fiberbot’s design comprised a hollow polymer cylinder and four pairs of equidistantly spaced resistive wires embedded around the cylinder’s circumference. The flow of electric current through these wires generates heat (Joule effect), leading to a localized increase in the temperature of the enveloping polymer and its combined thermal expansion (movie S1). The four pairs of wires facilitate the thermal expansion of the cantilever-like structure of the fiberbot and the bending of the fiber’s tip along its perpendicular plane, whereas the hollow-core configuration provides a means to introduce other diagnostic and therapeutic tools, reduces the fiber’s overall material (to be heated), and introduces a thermal barrier, thus enhancing the dynamic range and response of the device itself.

To optimize the fiber’s cross-sectional structure, finite element analysis was performed during the design process. Detailed simulation settings are described in note S1. In the simulation environment, the desired inner diameter of the fiberbot was set to a minimum of 1 mm based on the OD value of commercially available low-profile fiber-based diagnostic and therapeutic instruments. The effect of wall thickness was first explored as demonstrated in note S2, fig. S1 and table S3. The results indicated a 50% reduction in wall thickness (from 0.5 to 0.25 mm) followed by a 0.91-mm increase (30.4% increment) in fiber’s tip displacement, with an approximately 12.4°C rise (30% increment) in maximum temperature. In addition, we explored the impact of different wire locations within the cylindrical wall of the fiberbot and whose findings are detailed in fig. S2 and table S4. In terms of tip displacement, as the wires approach the central axis of the fiber, the magnitude of the displacement increases (by around 0.38 mm). After determining the best structure and material disposition using the previous simulations, we performed a final set of simulations with the target parameters for the fiberbot before translation into a physical device (fig. S3 and movie S2).

For the fabrication of the fiberbot, a scalable fiber drawing process was used to draw polymeric fibers from three-dimensional (3D) printed preforms (Fig. 2, A and B). This process facilitates the rapid and low-cost production of these high-aspect ratio materials with arbitrary cross-sectional designs (40 mm in OD, 24 mm in inner diameter, 2 mm in lateral through holes, and 200 mm long). Comparing different 3D printable and thermally drawable thermoplastics (table S5), we identified polycarbonate (PC) as a suitable candidate for the polymeric constituent of the fiberbot [Tg = 112° to 113°C, coefficient of linear thermal expansion α = 2.1° × 10^−5^°C^−1^, isotropic thermal conductivity = 0.22 W/(m·K)]. In addition to its relatively high thermal expansion coefficient and low thermal conductivity, PC has biocompatible grades, high mechanical strength, and dimensional stability, making it an ideal candidate for producing medical devices.

When defining the fiberbot’s OD, an upper limit of 2 mm was set to facilitate the insertion of the fiberbot through the working channel of commercially available endoscopes and laparoscopes. Therefore, the OD of the fiber was tuned between 1.65 and 2 mm by varying the draw speed (i.e., speed with which the fiber is pulled, *vd* = 0.4 to 0.6 m/min) at a constant down-feed speed (the speed with which the preform is fed into the furnace, *vf* = 1 mm/min) while monitoring the OD using a laser micrometer. Simultaneously, eight resistive wires (44 gauge, 50-μm-thick stainless-steel wires) were cofed through the 2-mm side channels of the preform and then passively pulled by the encircling polymer as the channels inside reduce in size (Fig. 2C). The 2-mm OD fiberbot, drawn from a preform with a 4 cm in diameter, presented a scale down ratio of 20. The inner diameter is expected to scale similarly, bringing the preform lumen of 2.4-cm down to a fiber lumen of 1.2 mm. The inner diameter of this fiber is 1.15 mm, deviating 4.2% from the expected value.

After drawing, the fibers were cut to desired lengths (10 to 15 cm), and the resistive wires were exposed at both ends by incising the fiber’s outer surface and pulling out the polymeric segment. The exposed wires at the fiber’s distal end were intertwined in pairs, and the conductive silver paint was applied to bond each pair and ensure reliable electrical contact. Kapton tape was then used to isolate and insulate the four wire pairs. The wires at the proximal end of the fiber were connected to a computer-controlled electronic circuit to directly impose voltage levels along the wire pairs. As an example, Fig. 2G demonstrates an overlay of the thermal images of the PC fiber’s planar bending (OD of 1.65 mm and length of 12 cm) upon the application of 12 V (dc) mode across a single pair of wires. In response to a temperature gradient of Δ*T* ≈ 20°C developed along the fiber’s cross section for a period of 3 s, the tip of the fiber translated approximately 7 mm (left and right) from the equilibrium position (movie S3).

### Fiberbot characterization

**Fig. 3.**
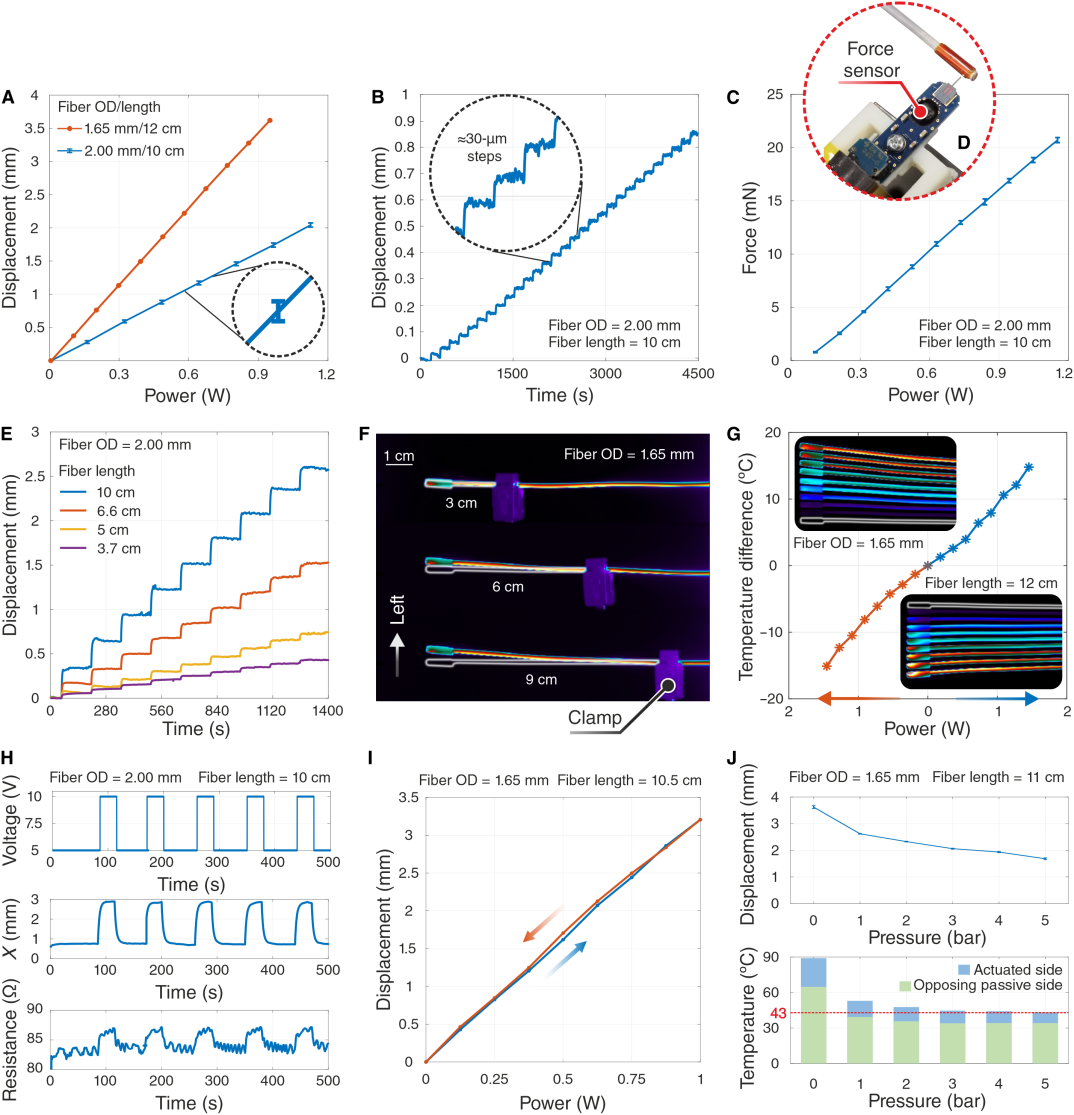
Mechanical characterization of the fiberbots. (**A**) Fiber tip displacement as a function of the applied power for two fibers with different ODs and lengths. Inset: Error bar indicating a SD (0.028 mm) obtained from five measurements using the laser displacement sensor. (**B**) Achieved positional resolution for incremental input power steps of approximately 13 mW. Inset: Zoomed-in view of the tip displacements. (**C**) Contact force generated laterally by the fiber tip as a function of the applied power (average of five runs). (**D**) Commercial force sensor used in the experiment. (**E**) Tip displacements for different unconstrained fiber lengths. (**F**) Top view of the thermal images of the fiber movement during the clamp testing. (**G**) Temperature gradient measured as a function of the applied power for displacements. (**H**) Waveforms for the input voltage (top), the corresponding tip displacement (middle), and the change in resistance of the embedded stainless-steel wires (bottom). (**I**) Hysteresis for a fiber is measured by performing an input power sweep from 0 to 1 W in 125-mW incremental steps. (**J**) Effect of the compressed air through the fiberbot’s central channel (at 38° ± 1°C environment). The top figure demonstrates the change in fiber tip displacement, and the bottom figure shows the reduction in the surface temperature of the fiber, as the air pressure is increased.

The fabricated fibers were further characterized in a well-controlled environment by investigating the relationships among power, displacement, length, temperature difference, and force (Fig. 3). The bespoke setup included manual positioning stages for geometry alignment, a laser displacement sensor, and an optical microscope focusing on the fiber’s distal tip to acquire accurate 2D deflection (position) information (fig. S4). From the same draw, using different draw speeds, two fiberbots with 1.65- and 2.00-mm OD and similar lengths (10 to 12 cm) were used for the subsequent characterization, with a commercial CO_2_ laser fiber placed inside the working channel of the fiberbot to simulate a real clinical scenario. From the obtained results, the two fiberbots exhibited a linear relationship between input electrical power and tip displacement (Fig. 3A), with the 1.65-mm OD fiber reaching a larger displacement (by a factor of 2.1) for the same input power level. Moreover, the behavior of the 2.00-mm OD fiber was repeatable across five runs with a pooled SD of 27 μm. Using the same characterization technique of powering only a single pair of wires at a time, we obtained the corresponding power versus displacement curves for the remaining three directions of motion. As shown in Fig. 3B, we demonstrated control of motion steps for the 2.00-mm OD fiber down to 30 μm over a dynamic range of 1 mm.

By its turn, the force generated (at zero displacement) by the tip of the same fiber was repeatedly reported linear with respect to the input power level and within the range of tens of millinewtons (20.7 mN at ≈1.153 W), i.e., up to seven times the fiber’s weight (3.1 mN) (Fig. 3C). Because the device’s length affects its response to thermal expansion, we examined this effect by mechanically clamping the fiberbot at different points (fig. S4B). The shorter (3.7 cm) free end of the 2.00-mm OD fiber produced smaller displacement (by a factor of ^1^/_6_) for the same input power of ≈1.446 W (Fig. 3E). Thermal images were also captured to visualize better this effect on the 3-, 6-, and 9-cm free ends of the 1.65-mm OD fiber (Fig. 3, F and G).

Given that the resistance of conductive materials (stainless steel) is a function of temperature, it was also important to characterize its influence during fiber actuation. We measured both the voltage and current signals on the actuated wire pair, thereby calculating the resistance of the wires in real time. A small resistance change of 2.8 ohm (≈3.5%) was recorded using differential amplifier circuitry (fig. S5) and repeatable across five cycles with 10-V input steps (average tip displacement of 2.1 mm; Fig. 3H). The 1.65-mm OD fiberbots also exhibited a small hysteresis of 87 μm within the actuation range of 3.21 mm, which makes them well suited for applications (e.g., robotics) that require precision over a continuous range (Fig. 3I). However, as the surface temperatures may exceed 80°C, we introduced active cooling and characterized the effect of the cooling effect on tip displacement. We placed the fiberbot with the laser displacement sensor inside an incubator at 38° ± 1°C to simulate the human body temperature and examine the active cooling process by injecting compressed air through the central channel of the fiberbot (fig. S6). Our experiments show that by using air at room temperature, we can decrease the surface temperature rapidly with a small reduction in fiber tip displacement at low pressures. Further increase of flow pressure presents an incremental decrease in temperature but a substantial reduction in tip displacement (Fig. 3J). Actual assessment of tissue damage at various temperatures and conditions is further demonstrated in other sections of the current manuscript during the in vivo animal testing.

Last, we assessed the velocity profiles (starting from rest) for different input voltage steps applied to the 2-mm OD fiberbot (fig. S7A). The velocities exponentially decreased as the tip approached the steady-state position with an average time constant of τavg = 1.4 s and maximum achievable velocities varying from 0.008 to 0.8 mm/s for voltage levels between 1.118 and 10 V (fig. S7B). Furthermore, the average time required by the fiberbot to move from 10 to 90% of its fully developed steady-state position (i.e., rise time) was measured at 8.6 s (fig. S7C).

### Fiberbot control

**Fig. 4.**
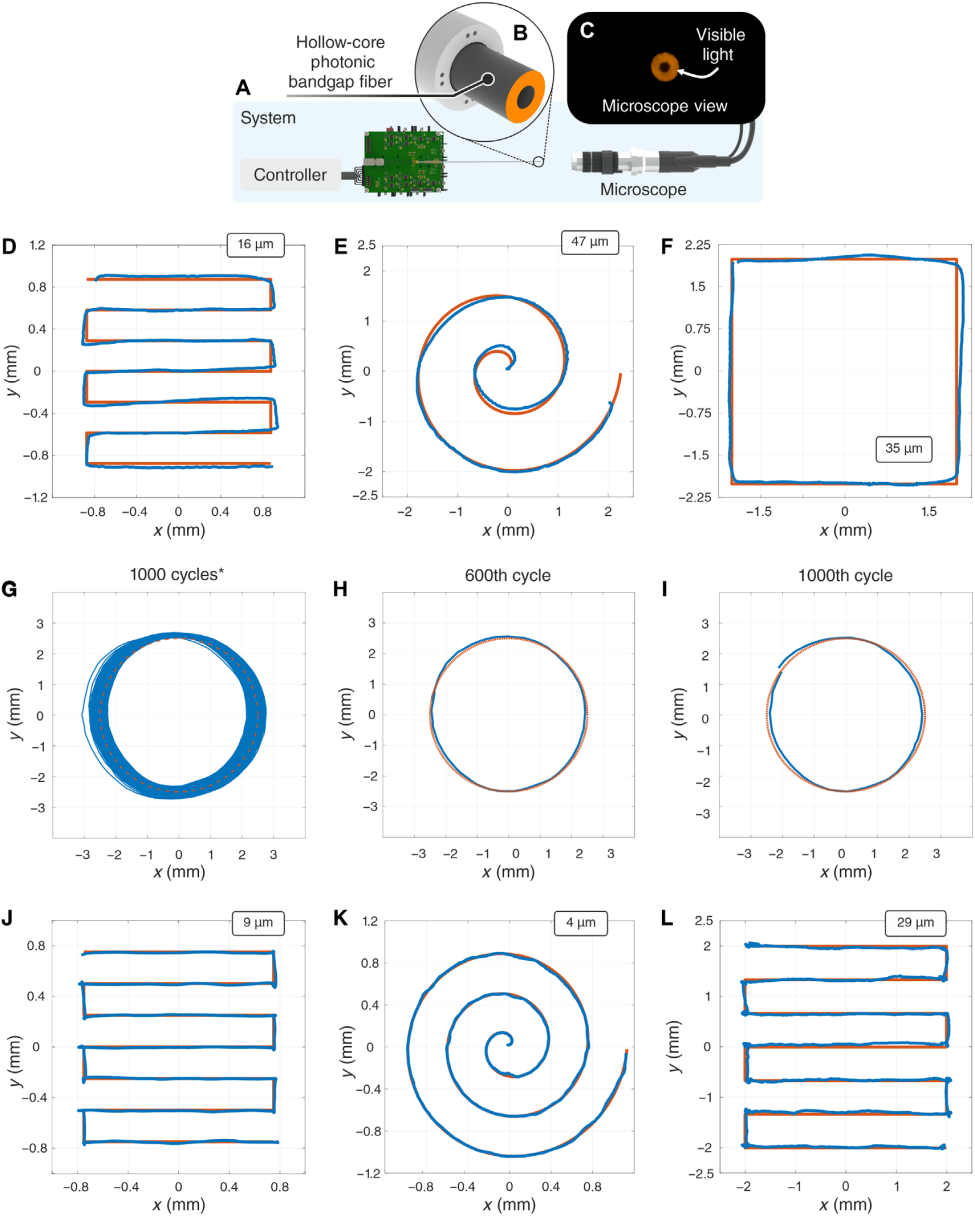
Control of the fiberbot motion and further characterization. (**A**) System used to track the fiber tip displacement. (**B**) Embedded 900-μm hollow-core PBG fiber connected to a 650-nm LED for tracking purposes. (**C**) Microscope view of the embedded fiber. (**D** to **F**) Measured (blue) versus desired (orange) paths based on several manually tuned inputs (D) raster [184 s], (E) spiral [42 s], and (F) square [97 s]. (**G**) Fiberbot was commanded to follow a circular path for 1000 cycles without losing their target performance characteristics. (**H** and **I**) Detail of the circular path for cycle numbers 600 and 1000, respectively. (**J** and **K**) Measured (blue) versus desired (orange) paths based on the vision-based feedback approach [raster: 244 s, spiral: 641 s]. (**L**) Measured (blue) versus desired (orange) paths based on the temperature-based feedback approach [737 s].

To accurately track the motion of the fiberbot, we embedded a hollow-core photonic bandgap (PBG) fiber coupled to a 650-nm light-emitting diode and used an optical microscope to monitor the position of the PBG fiber tip (Fig. 4, A to C). The fiberbots were evaluated by commanding them to follow several predefined desired trajectories, which comprised a set of points on the projected orthogonal plane in front of the fiber tip (light spot). For the open-loop control, these points were mapped into four independently controlled and timely tuned voltage inputs for the pairs of wires composing the fiberbot. These mapped voltages were then sequentially sent from the computer to the electronic interface, enabling precise control over the fiberbot’s motion. Subsequently, we used an iterative compensation technique (see note S5 for detailed explanation and fig. S8) to optimize the accuracy of control and reduce positional errors measured along raster, spiral, and square paths (Fig. 4, D to F). The average path errors (i.e., the time-independent difference between the desired and measured paths) were reported to be lower than 50 μm for the tested trajectories.

To determine the durability extension of the fiberbot and its ability to maintain targeted performances, we commanded the fiberbot to follow a circular path for 1000 cycles with open-loop control mode (Fig. 4, G to I). A positional deviation of less than 0.7 mm was observed among the overlapped circle cycles, with each cycle maintaining consistent similarity in terms of dimensions and circularity. In addition, we further characterized the maximum achievable speed of the fiberbot while performing circular trajectories with minimum acceptable positional errors (fig. S9). Within these conditions, the maximum achievable speed was found to be 10.3 mm/s when the average errors are restricted to 80 μm.

In addition to using open-loop control, we explored other control capabilities of the fiberbot in the form of closed-loop mechanisms. This was initially achieved by using an optical-based approach using the tracked tip position in real-time as parameter feedback (fig. S10A). The results shown in Fig. 4 (J and K) demonstrate the fiberbot navigating through raster and helical paths with average path errors of less than 10 μm when using closed-loop control. We then implemented a temperature-based feedback control mechanism to remove the optical system in front of the fiberbot (and thus be closer to a real surgical scenario). This involved attaching four miniature thermocouples to the outer surface of the fiberbot and positioned alongside the four wire pairs (fig. S10B). By leveraging the outcomes of previous characterization results, we were able to map the desired tip displacement to the corresponding temperature difference between opposing sides of the fiberbot. These desired temperature differences were then used as setpoints for the feedback control (as detailed in fig. S10C). For a raster trajectory of 4 mm by 4 mm, the fiberbot could follow the desired path with an average path error of 29 μm (Fig. 4L).

### Fiberbot with endomicroscopy

**Fig. 5.**
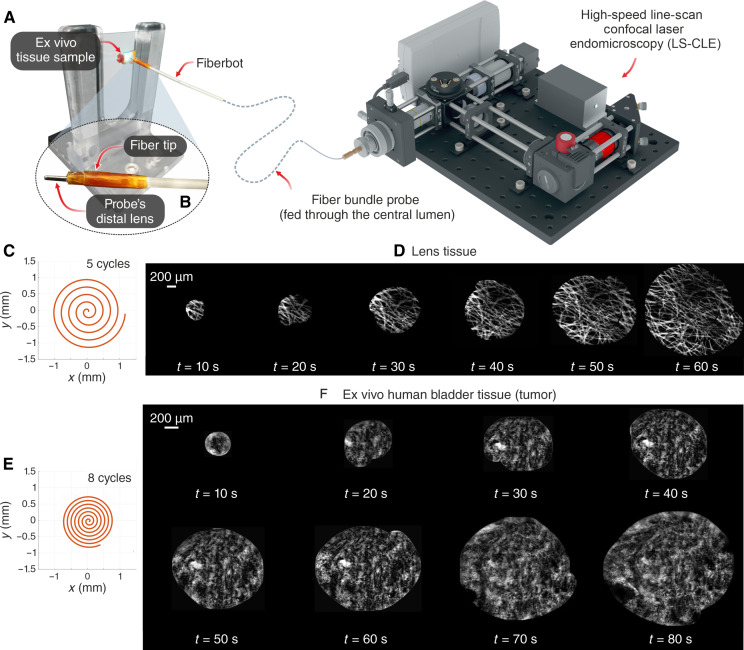
Integration with the LS-CLE platform. (**A**) Overview of the integration between the LS-CLE system and the electrothermal fiber via a fiber bundle probe. (**B**) Close-up image of the thermally drawn fiber embedded with a probe through its central channel. (**C** and **E**) Desired spiral path of five and eight cycles to be followed by the fiber. (**D** and **F**) Growing mosaics reconstructed at every 10-s interval from the acquired images while scanning a lens tissue and an ex vivo human urothelial carcinoma tissue, respectively.

The performance and control of the fiberbots explored in the previous sections suggest many possible applications in MIS. Figure 5A shows an exemplary application where we deployed the fiberbots to precisely maneuver an imaging fiber bundle probe, a flexible tool from several endomicroscopic imaging systems used by surgeons and pathologists to intraoperatively detect and distinguish between cancerous and healthy cells with surgical margins. This integration addresses the challenge in imaging larger tissue areas given the fiber bundle’s relatively small FOV (325 μm) by moving the probe inside the fiberbot over an area of several square millimeters, with the individual frames being stitched together in quasi real time to form a continuous mosaic image (*50*).

To implement this, we inserted the fiber bundle (0.9 mm in OD, 3.5-μm resolution) through the fiberbot’s central channel (Fig. 5B) and optically coupled its proximal end to a portable and compact probe-based confocal laser endomicroscopy (pCLE) system. The probe was then moved at its distal end along a preoptimized spiral path at a scan time of 40 s (lens tissue) and 140 s (ex vivo tissue from the urinary tract surface) (movie S4). On the basis of the growing mosaics reconstructed every 10 s from the acquired images, the integrated device was able to yield contiguous mosaics with high image quality. In the lens tissue case, the hyperfluorescent fiber structures were visible throughout the scan area (Fig. 5, C and D) whereas, for the urothelial carcinoma tissue (and despite it being a soft surface), the large-area mosaic was reconstructed without any gaps, showing features such as hyperfluorescent clusters of nuclei with no defined organization and fibrovascular stalks with a distorted vasculature (Fig. 5, E and F). These preliminary results demonstrate the feasibility of obtaining histology-like images with the fiberbot without removing or destroying any structure located in large tissue areas.

### Fiberbot with surgical laser and mass spectrometry

Our next practical demonstration focused on presenting the fiberbots’ potential to combine with other standard medical instruments to perform a surgical procedure requiring cellular-level precision. We coupled the fiberbot with a therapeutic tool (a surgical CO_2_ laser source) and a diagnostic tool [rapid evaporative ionization mass spectroscopy (REIMS)] to showcase precise tissue ablation and identification of a 10-μm-thick slice of mouse brain tissue (*9*)*.* REIMS is a mass spectrometry technique originally developed for the identification of biological tissue during surgery, making use of electrosurgical instruments to ablate tissues, with the resulting aerosol being collected by a mass spectrometer to analyze its chemical composition. REIMS systems have been previously used for the screening and diagnosis of colorectal, cervical, and endometrial cancers (*51**–**53*), with the recent variant of laser desorption–REIMS (LD-REIMS) ablating tissues in narrow areas with higher spatial precision and easier data collection (*54*, *55*)*.*

**Fig. 6.**
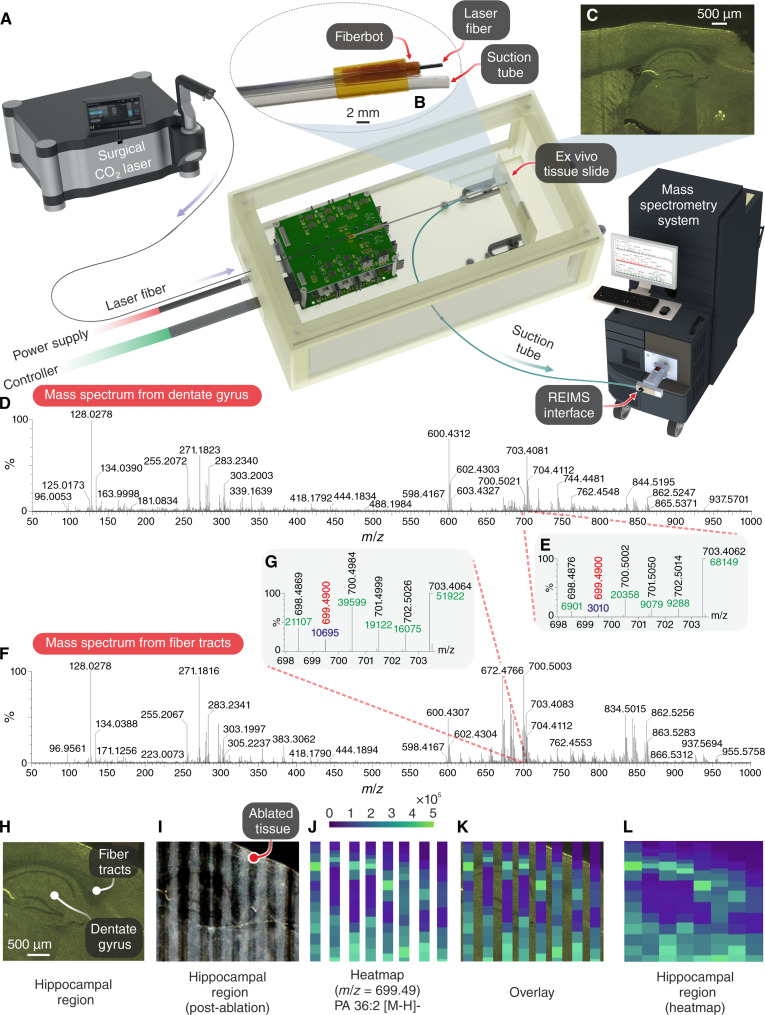
Integration with the REIMS platform. (**A**) Overview of the integration between the surgical CO_2_ laser, electrothermally actuated fiber, and REIMS interface. (**B**) Close-up image of the thermally drawn fiber embedded with a laser fiber through its central channel and an externally attached smoke collection tube. (**C**) Optical image of a mouse brain’s sagittal section of the hippocampal region. (**D** and **F**) Mass spectra corresponding to the dentate gyrus and fiber tracts, which show a difference in the ion species and intensities corresponding to the gray matter distribution in the former and white matter in the latter. (**E** and **G**) Close-up of the mass/charge ratio (*m*/*z*) 699.4900. (**H**) Cropped optical image of the hippocampal region. (**I**) Optical image following the laser ablation of the tissue sample (parallel ablated lines created by moving the laser fiber using the electrothermally actuated fiber). (**J**) Single-ion image heatmap for *m*/*z* 699.4900 tentatively assigned as phosphatidic acid PA 36:2 [M-H]- structure, showing abundance in the fiber tract and relative absence in the dentate gyrus. (**K**) Overlay of heatmap onto the optical image of the hippocampal region. (**L**) Stitched heatmap.

First, we introduced the previously used flexible PBG laser fiber through the working channel of the fiberbot to ablate ex vivo tissue samples and attached a silicone suction tube to the outer surface of the fiberbot to collect the generated aerosol for analysis by the LD-REIMS system (Fig. 6, A to C). The tissue samples were ablated by autonomously moving the embedded laser fiber along a preoptimized path consisting of parallel lines at an average speed of 0.7 mm/s and scan time per line of 6.3 s, during which data were collected through the suction tube at a rate of 5 scans/s (movie S5). Figure 6 (D to G) demonstrates the mass spectra obtained from the tissue regions of the dentate gyrus and fiber tracts within the mouse brain, respectively, revealing a comprehensive molecular coverage of small molecules, fatty acids, and structural phospholipids.

For better visualization of the medical imaging capabilities of the integrated system, we then generated a heatmap image of the phosphatidic acid PA 36:2 [M-H]- structure from the acquired using data, which was afterwards aligned and overlaid onto the optical image of the tissue sample (Fig. 6, H to L). The green-yellow bars represent a higher abundance, whereas the purple regions hint at the absence of this molecule, thus showing a corresponding overlap with the fiber tracts region of the mouse brain that correlates well with the a posteriori histological examination, in opposition to the low-intensity areas (dentate gyrus). The fiber tracts are typically white matter regions composed of myelinated axons, while the dentate gyrus is composed of gray matter made up of cell bodies: This explains the difference in mass spectra observed between these two regions, as the biochemical composition varies in both ion species and relative intensities. Hence, the high-precision motion of the fiberbot coupled to REIMS can help identify different types of tissues with spatial resolution well above the millimeter level, only limited by the spot size of the laser beam in this case.

### In vivo validation of the fiberbot with a porcine model

**Fig. 7.**
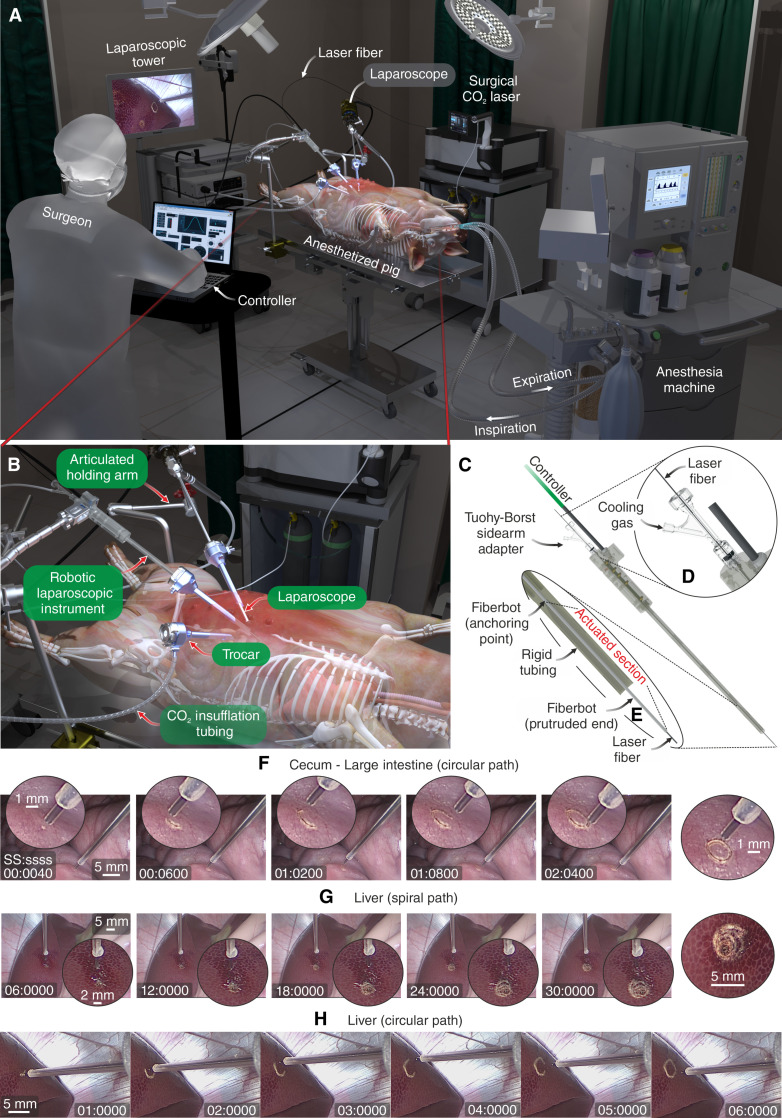
In vivo demonstration of the laparoscopic robotic instrument in a porcine model. (**A**) Graphical overview of the experimental setup. The setup features an endotracheally intubated pig put under general anesthesia in the supine position, an anesthesia machine used to induce safe apnea phases (i.e., suppress respiratory-induced motion) during the robotically controlled ablations, a surgical CO_2_ laser source that is coupled to the flexible PBG laser fiber, a laparoscopic tower that is optically coupled to the laparoscope, and a control unit that the surgeon teleoperates the fiberbot. (**B**) Close-up view of the CO_2_ insufflated abdominal cavity, inserted trocars, and the subsequently advanced laparoscope and robotic instrument. The articulated arms are used to position, orient, and secure the laparoscope and robotic instrument in place. (**C**) Illustration of the robotic instrument’s handle, which hosts the electronics interface and the rigid tubing acting as a conduit for the fiberbot. (**D**) Close-up view of the Tuohy-Borst adapter, which facilitates the simultaneous insertion of the laser fiber and injection of cooling gas through the fiberbot’s central channel. (**E**) Cross-sectional view of the actuated section of the fiberbot and the rigid tubing’s distal end. (**F** to **H**) A photographic time series (captured using the laparoscope) of the fiberbot's simultaneous actuation and ablation (during the safe apnea phases) of a (F) circular path in the cecum, (G) spiral path in the liver, and (H) circular path in the liver. Note that the scales were estimated using the closest point of the laser fiber to the tissue.

The series of demonstrations presented previously have showcased the fiberbot’s capabilities in providing high-precision fiber motion, well-controlled ablation, and high-resolution tissue imaging. To demonstrate that the precision of the fiberbot can be delivered safely under realistic in vivo conditions, we conducted an animal trial with a porcine model (Fig. 7, A and B). We developed a laparoscopic robotic instrument using the proposed fiberbot (Fig. 7, C to E) to safely deliver precise tissue ablations during single-port, multi-quadrant surgeries. In this exemplar, the instrument was used to access the liver and intestine within the abdominal region. The standalone instrument comprised an electronic interface inside the handle portion, onto which the resistive wires of the fiberbot are soldered and anchored. The fiberbot being anchored to the instrument ensures the robustness of the system during the animal trail. Attached to the instrument’s handle is a 35-cm-long rigid tube that acts as a conduit for the fiberbot that protrudes 35 mm out of the tube. The 3D printed rigid tube and the handle are printed in two halves lengthwise, enabling the fiberbot and its relevant electronics interface integrated inside one half of the structure. The tube’s inner diameter matched the OD of the fiberbot, so that the fiberbot was held tightly inside the rigid tube, only allowing the distal section of the fiberbot to be actuated, ensuring that the fiberbot’s performance was not altered. At the proximal end of the handler lies a Luer adapter that connects a Tuohy-Borst side-arm adapter to the fiberbot. The Tuohy-Borst adapter facilitates the simultaneous (i) insertion of the flexible PBG laser fiber through the working channel of the fiberbot, (ii) injection of helium gas through the small gap between the laser fiber and the fiberbot to cool down the fiberbot’s average temperature, and (iii) prevention of backflow from the injected helium gas.

Before the animal trial, two experiments were performed with the described laparoscopic fiberbot. First, to evaluate the influence of the integrated instruments on the motion performance, we tested the laparoscopic fiberbot deflection with and without surgical instruments inside the working channel. This experiment involved applying different voltage levels to the laparoscopic fiberbot without the presence of instruments (hollow core) and with the imaging fiber bundle or CO_2_ laser fiber. From fig. S11, a consistent quadratic relationship between the fiberbot displacement and applied voltage levels is observed, irrespective of the inserted instrument, with a reported 20 and 13% displacement reduction for the CO_2_ laser fiber and the pCLE imaging fiber bundle, respectively. Second, an electrical safety test for this integrated device was performed (note S6), in which the protruding end of the laparoscopic fiberbot was immersed inside a container filled with a standard conductivity solution of 0.1413 S/m, a value that mimics the average electrical conductivity of the tissues located within the abdominal region (fig. S12). It was found that no leakage currents circulated from the laparoscopic fiberbot to the surrounding conductive solution, thus assuring proper electrical current isolation of the instrument for the subsequent animal experiment.

During the in vivo experiment, the pig was endotracheally intubated and put under general anesthesia in the supine position. The trial commenced by inserting an 11-mm-long trocar into the abdomen through an incision made along the umbilicus. The bladeless obturator of the trocar was then removed, and CO_2_ gas (15 mmHg at a rate of 4 to 6 liters per minute) was insufflated into the abdominal cavity (pneumoperitoneum) through the trocar to create an adequate workspace between the viscera and abdominal wall. A laparoscope was then advanced through the trocar sleeve to visually guide the insertion of two additional 11-mm-long lateral trocars. Once all trocars were inserted, their bladeless obturators were removed, and the robotic instrument was introduced through one of the two trocar sleeves under the guidance of the laparoscope. Subsequently, both instruments (i.e., laparoscope and robotic instrument) were positioned, oriented, and secured in place using the locking mechanism of the articulated holding arms. Note that, in some cases, the laparoscope and robotic instrument are retracted and inserted through different trocars for better access and visualization of the target tissue.

Before actuating the fiberbot and performing ablations on the target tissue, we had to mitigate the inaccuracies introduced by the movement of abdominal organs caused by mechanical ventilation [i.e., respiratory-induced motion (RIM)]. For example, during mechanical breathing, the liver’s RIM could range from 8 to 25 mm in one direction (*56**–**58*). Therefore, if RIM is left unsuppressed, then our instrument could lose its accuracy and precision due to the time-varying offset between the fiberbot’s focus and the target tissue. Thus, making it challenging to locate and target exact lesions without risking damage to the surrounding healthy tissue (*59*)*.* Researchers over the years have developed machine learning, computer vision, and advanced control techniques to track and compensate for target tissue motion using teleoperated (*60*, *61*) and autonomous robotic platforms (*62*)*.* Although these approaches have proven their feasibility and effectiveness in the compensation of RIM, the focus of this study was to demonstrate the potential of the laparoscopic fiberbot as a standalone instrument without introducing additional equipment and complexity to surgeries.

To achieve this objective, we pursued a well-established and safe intraoperative technique called “adequate preoxygenation with apneic oxygenation.” This method uses the anesthesia machine to control respiratory movements during procedures that require higher precision [e.g., radiation therapy in breast cancer treatment (*63*) and coronary artery bypass grafting (*64*, *65*)]. In this technique, anesthesiologists start by preoxygenating (denitrogenating) the patient to 100% oxygen (O_2_) for a few minutes before switching off the mechanical ventilator and inducing a long and safe apnea phase (i.e., periods with no breathing and RIM in intubated patients). During the apnea phase, the patient’s O_2_ flow is maintained while the physician performs the desired treatment. Anesthesiologists preoxygenate the lungs to increase the O_2_ reserve within the alveoli, thus increasing the safe apnea time. Otherwise, the persistence of nitrogen and the accumulation of CO_2_ during apnea could speed up the onset of hypoxemia (i.e., lower levels of oxygen in the blood than normal). The safe apnea time, i.e., the duration until critical arterial desaturation occurs following the cessation of ventilation, can be up to 8 min in healthy adults, 5 min in moderately ill adults, and 2.7 min in obese adults (*66*)*.* Critical arterial desaturation occurs when O_2_ saturation (SaO_2_) falls below the upper inflection point on the oxygen-hemoglobin dissociation curve, beyond which a further decrease in the partial pressure of O_2_ (PaO_2_) results in a rapid decrease in SaO_2_. Note that SaO_2_ is the percentage of oxygen in the arterial blood, PaO_2_ is the oxygen pressure in the arterial blood, and the oxygen-hemoglobin dissociation curve is the curve that correlates oxygen saturation across a range of oxygen pressures. Moreover, the timeframe of minutes is adequate to execute the desired laparoscopic ablations using the fiberbot (*67*)*.* In addition, we monitored the pig’s physiological variables (i.e., SaO2, heart rate, temperature, inspiratory CO_2_, end-tidal CO_2_, inspiratory O2, expiratory O2, and inspiratory and expiratory inhalational anesthetic agents) during the experiments to ensure and demonstrate the safety of apneic oxygenation.

After identifying different target organs and tissues to ablate, our instrument’s efficacy was evaluated during the apnea phase by commanding the fiberbot, with the flexible CO_2_ laser fiber introduced and activated (and helium gas injected at 50 psi), to follow several predefined desired paths (e.g., circle and spiral). Figure 7 (F to H) shows three photographic time series of the laparoscopic fiberbot following different paths while it simultaneously and safely ablates the cecum (part of the large intestine) and liver tissue. For the cecum, the ablation of the circular path took approximately 2 s with no additional complications or changes in the pig’s physiological condition verified. Similarly, for the liver, the fiberbot took 30 s to complete the spiral path and 6 s to complete the relatively larger circular path, both with no adverse events (e.g., critical arterial desaturation and CO_2_ accumulation) observed during and after the experiments (movie S6).

### Validation of fiberbot thermal safety in vivo

**Fig. 8.**
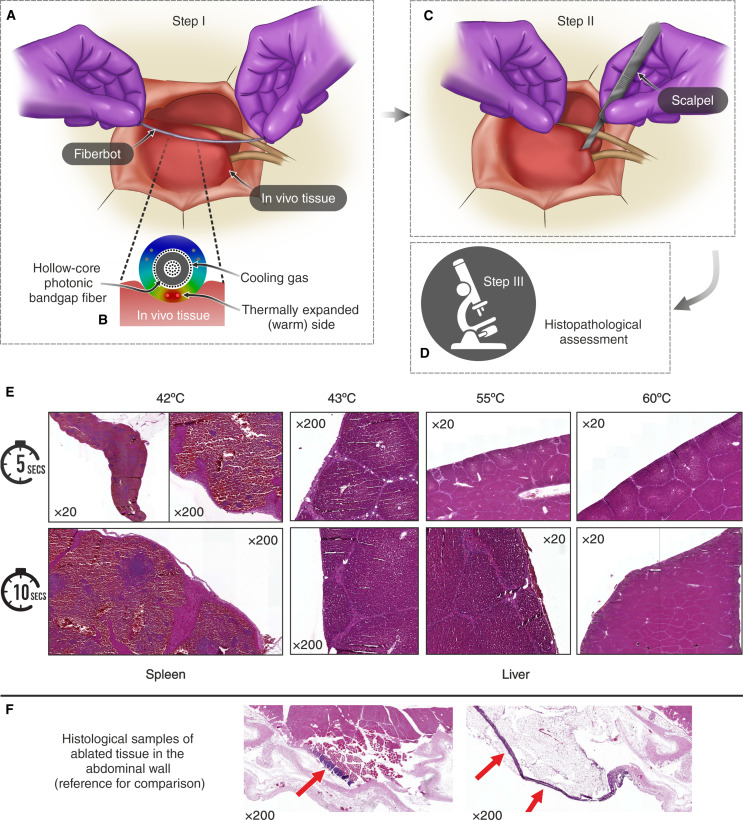
Histopathological assessment of thermal injury. (**A**) Graphical illustration of the first step in the experiment, where the actuated side of the fiberbot is held against the in vivo tissue for a few seconds. (**B**) Cross-sectional closeup view of the fiberbot and its interaction with the in vivo tissue. The fiberbot is embedded with the flexible PBG laser fiber and injected with a constant pressure of helium gas to cool it down. Note that the laser fiber uses a similar cooling technique using helium gas. (**C** and **D**) Graphical illustration of the second and third steps in the experiment, where the exposed tissue is excised for the histopathological assessment of thermal injury. (**E**) Optical images of eight 4-μm-thick and hematoxylin and eosin (H&E)–stained spleen and liver tissue slides. The acquired tissue samples were exposed to different temperatures (42°, 43°, 55°, and 60°C) for differing durations (5 and 10 s). No morphological signs of coagulation due to thermal injury were observed at the capsular surface or parenchyma of the liver and spleen tissue samples. ×20 and ×200 indicate the magnification of the objective lens used to capture these images. (**F**) Optical images of two ablated tissue samples of the abdominal wall which demonstrate thermal injury of connective and muscle tissue at the surface of the peritoneum (i.e., serous membrane forming the lining of the abdominal cavity). These examples are presented as a reference for comparison with our experimental results.

Under the same in vivo conditions, we demonstrated the thermal safety of the instrument by conducting a series of experiments where the fiberbot was actuated (i.e., one side of the tube is heated) and held against the in vivo tissue for a few seconds. During these experiments, we kept the (i) flexible PBG laser fiber advancing through the central channel of the fiberbot, and (ii) helium gas injected at a constant pressure of 50 psi to cool down the fiberbot. Using a thermocouple, we monitored and adjusted the outer surface temperature of the fiberbot by controlling the (electrical) input power. We tested four different temperatures (42° ± 1°C 43° ± 1°C, 55° ± 1°C, and 60° ± 1°C) and held the heated side of the fiberbot against the liver and spleen tissues for two different exposure times (5 and 10 s) as shown in Fig. 8 (A to D). The exposed tissues were subsequently excised for histopathological assessment of thermal injury, with the posterior analysis showing no morphological signs of coagulation due to thermal injury in all tissue planes of the liver and spleen (Fig. 8, E and F). Therefore, these results validate the efficacy and safety of the fiberbot in realistic in vivo conditions when deployed as a robotic laparoscopic instrument.

## DISCUSSION

In this work, we presented a fiber robot that can move with high precision by asymmetric thermal expansion of its enveloping polymer and induced by the flow of electric current through longitudinally embedded resistive wires (Joule heat). The rapid prototyping approach we developed simplifies the production of hundreds of meters of inexpensive, bespoke, precise, and integrable fibers that can readily function as flexible and compact polymer-metal fiberbots or actuators. Unlike previous research efforts that use thermally responsive polymers to steer tubular structures [e.g., hydrogels that shrink (*68*) and liquid crystal elastomers that change in phase (*69*) when heated], we functionalized a class of thermoplastic polymers that are often (i) not classified as thermo-responsive polymers and (ii) disregarded in the actuation of soft robots due to their relatively large flexural rigidity (i.e., the stiffness of the material when its subjected to bending) when compared to softer polymers. Moreover, we capitalized on their stiffness and intrinsic (positive) thermal expansion property to create stable actuators that can omnidirectionally move with high precision and negligible hysteresis. Although PC and stainless-steel wires were used in our study, the findings can be reproduced using thermally drawn fibers with different cross-sectional designs made of similar thermoplastic polymers [e.g., polysulfone, polyetherimide, poly (methyl acrylate)] and metallic wires (e.g., nickel-chromium and nickel-titanium).

After characterizing and controlling the millimeter-scale fiberbots, we presented several demonstrations targeting clinical applications that demanded cellular-level precision from high-aspect ratio devices. The fiberbot’s tubular structure facilitated these demonstrations by seamlessly integrating therapeutic and diagnostic platforms, thus providing high-precision manipulation, ablation, and enhanced tissue analysis in situ down to the cellular level. The pCLE scan providing a subcellular resolution image of a larger tissue area demonstrates the potential to help surgeons visualize and assess abnormal cells, critical structures, or surgical margins. The trajectories ablated by surgical laser showcase the ability to work precisely near critical structures or reduce the damage to surrounding healthy tissue. The high resolution of the molecular map acquired in conjunction with the REIMS system demonstrates the potential reduction of surgical margins from several millimeters down to a fraction of a millimeter.

The use of the fiberbot as a laparoscopic instrument during the animal trial demonstrated the feasibility of the in vivo use of this technology. In addition, the subsequent histopathological assessment showed that although the fiber is electrothermally actuated and can reach high outer surface temperatures (up to 60°C with helium gas injected), it does not cause any thermal injury to the surrounding tissue for exposure times of up to 10 s. The elevated temperatures did not present any safety concern due to the low thermal mass of the miniature fiberbot and due to the low thermal conductivity of the encapsulating polymer of the fiber that acts as a buffer for heat transfer, limiting the amount of heat transferred to surrounding tissues. Moreover, our electrical safety experiments corroborate that the fiberbot is safe and reliable for use in vivo. Therefore, we envisage that the proposed instrument can give surgeons greater control in minimally invasive surgeries.

Nonetheless, there are some potential technical challenges to consider for further clinical translation of the fiberbot. First, the fiberbot’s length affects its response to thermal expansion, i.e., the shorter its dimension, the smaller the achievable range of motion and workspace. Second, in clinical applications where the target tissue is located within confined and tortuous spaces, the relatively long actuated section of the fiberbot can impede it from being positioned normally to the target tissue. Last, although the embedded straight metallic wires expand, their thermal expansion is relatively smaller when compared to the enveloping PC, which also restricts the overall motion of the fiberbot. Integration of the fiberbot with complementary instruments, such as the articulated arm with the locking mechanism we demonstrated in the porcine study, commercial surgical robots, or other endoscopic instruments, may facilitate the delivery and coarse positioning of the fiberbot in the desired location.

However, the stability of the delivery platform is crucial, as instability can compromise the precision of the fiberbot, diminishing its main advantage. Thus, flexible endoscope systems, such as colonoscopes and gastroscopes, require a stabilization mechanism for fiberbot delivery. This could be achieved, for instance, by using an endoscopic system equipped with an integrated balloon at the tip of the conventional endoscope. This balloon, when moderately inflated, improves the stability of the endoscopic instrument (*70*), which presents the potential to cooperate with fiberbot. In addition, the collaborative approach of fiberbot directly with stable coarse positioning platforms can compensate for the lack of range of motion of fiberbot, expanding the potential for achieving precise surgical outcomes in constrained spaces. The fiberbot, for example, can be passed through a lumen of a Da Vinci instrument (potentially a suction irrigation instrument), or the lumen of laparoscopic instrument as demonstrated in the pig study, or it can also be mounted onto a colposcope. The design should also consider an anchoring mechanism at the distal end of the device that clamps the fiberbot leaving only the desired length free to move for actuation, as potential gaps between the fiberbot and the instrument channel may also influence the motion.

The proposed fiberbot presents the potential for radical improvements in the area of minimally invasive cancer surgery. When combined with diagnostic tools, the margins of a tumor, for example, could be mapped intraoperatively with high precision, leading to the sacrifice of only a fraction of a millimeter of healthy tissue, as opposed to several millimeters required in current surgical procedures for histopathological analysis. In addition to its precision, the long and low-profile structure of the fiberbot presents access and visualization benefits in challenging surgeries, such as vocal cord or cervical cancers and even cancers involving skin as they mostly appear in the head and neck region (*71*). Prior research introduced the Da Vinci robotic platform for manipulation of laser fibers during vocal cord cancer resection, which still necessitates improved access and submillimeter ablation margins (*72*). Similarly, addressing precancerous cervical lesions involves tissue excision or ablation, with an overall 23.1% rate of incomplete excisions resulting in positive margins, from which 17.1% still present risk of residual or recurrent issues (*73*). For these scenarios, the combination of our fiberbot with coarse positioning platforms and surgical lasers, imaging fiber bundle or LD-REIMS systems, can be added to the interventional picture, facilitating precise localization, molecular-level histology, and safe and complete removal of cancerous tissue. Our results provide a strong basis for developing a clinical tool combining robotics, diagnostics, and therapeutics, the basic components of an autonomous surgical system, which could perform accurate and molecular histology-guided surgical resections.

## MATERIALS AND METHODS

### Preform fabrication

The preform fabrication process commenced by defining the geometry of the preform using 3D computer-aided design (CAD) software (SolidWorks 2020, Dassault Systèmes, France). The preform design was then 3D printed by feeding PC (Ultimaker PC Transparent, 2.85 mm; Netherlands) filaments into a commercially available fused deposition modeling printer (Ultimaker 3 Extended, Netherlands). The warping of the 3D printed preform was prevented by: (i) using an adhesive layer (DIMAFIX, 3D GBIRE, Lancashire, United Kingdom), which was sprayed onto the glass build plate, and (ii) fitting the printer with an upper enclosure and a door (Accante cover Ultimaker 3 Extended, 3D GBIRE, Lancashire, United Kingdom) for a constant printing chamber temperature during the printing process. The dimensions of the 3D printed preform were 40 mm in diameter and 200 mm in length. The central channel was 24 mm in diameter, while the smaller side channels were 2 mm in diameter. For the 3D printing parameters, refer to table 1 in (*74*).

### Fiber drawing

The preform was coupled to a Polyether ether ketone (PEEK) 30% glass fiber reinforced preform holder (Ketron Peek glass fiber reinforced rod, 20 mm in diameter; Bay Plastics Ltd., UK) and a wire feeding platform comprising eight bobbins of 50-μm-thick stainless-steel wires [44 American Wire Gauge (0.05 mm), The Crazy Wire Company, UK]. Because the preforms are printed layer by layer and have lower tensile strengths than molded or extruded solid structures, their desired viscosity, when drawn, is expected to be lower than that of solid preforms (*75*). By setting a high initial draw temperature of 265°C, the preform’s viscosity dropped several orders of magnitude (fig. S13) and necked down under (i) the constant tension of 55 g and (ii) the weight of the lower end of the preform, which formed the neck-down region. Note that the actual temperatures inside the furnace (TV-05 Solid Tubular Furnace, The Mellen Company, US) are lower, refer to (*76*) for the furnace calibration. When the lower end of the preform exited the furnace, it was attached to a pulling system, and the draw temperature was lowered gradually to 220°C. This fabrication process is demonstrated in movie S7.

### Experimental setup for fiberbot actuation characterization

The enclosed setup in fig. S14 was built to provide a stable characterization environment for the fiberbot with minimal external interference. An XYZ stage (LT3/M-XYZ translation stage, Thorlabs, USA) was used to adjust the printed circuit board position for aligning the fiberbot’s tip with an optical triangulation laser sensor (LK-G5000, Keyence, Japan). This sensor was used to accurately measure the displacement of the fiberbot’s tip in a specific direction. With the characterization setup completely enclosed, the proposed fiberbot system’s resolution, accuracy, and repeatability were assessed. During all the characterization experiments, the fiberbot was embedded with a hollow-core PBG fiber (0.9 mm in OD, fiber was stripped of its polymeric jacket, OmniGuide, USA) to facilitate the tracking of its tip. This PBG fiber was coupled to a 650-nm light-emitting diode (696-1838, RS, UK) (light is guided by the polymer cladding of the PBG fiber), and a digital microscope (VHX-6000, Keyence, Japan) was used to track its motion in 2D. Images from the microscope were captured using an image grabber (AV.io HD, Epiphan Video, Canada) and processed with LabVIEW-based image tracking software (*77*)*.* The position of the PBG fiber tip was determined using the IMAQ Match Pattern algorithm of the NI Vision 2018 library (version 2018, National Instruments, USA), which provides the position of the steered PBG fiber in ***P***_**PBG_TIP**_ = [*X*_PBG_TIP_ and *Y*_PBG_TIP_] in each frame. After the first position is calculated, a region of interest around the PBG fiber tip is automatically generated (centered in ***P***_**PBG_TIP**_) to reduce the processing time and overall accuracy and reliability of the tracker.

In Fig. 3A, the relationship between input electric power and tip displacement was characterized by performing an input power sweep from 0 to 0.95 W in ≈95-mW incremental steps for the fiber with an OD of 1.65 mm and 12 cm in length and from 0 to 1.12 W (160-mW increments) for the fiber with an OD of 2.00 mm and 10 cm in length. The input power was maintained for each measurement for 2.5 min, and the average tip displacement of the last 1.5 min was calculated. For the (2.00 mm and 10 cm) fiberbot, the same experiment was repeated five times, and the mean and SD of each set of measurements for the same input power were plotted. In Fig. 3B, using the (2.00 mm and 10 cm) fiberbot, the positional resolution was measured by also performing an input power sweep with ≈13-mW increments, intermitted by 2.5-min intervals.

For the contact force measurements shown in Fig. 3C, the (2.00 mm and 10 cm) fiberbot’s tip was placed against a flat-tipped force sensor (FT-S1000000 μN, FemtoTools, Switzerland). The sensor was mounted on top of a 25-mm XYZ translation stage (PT3/M, Thorlabs, USA) and in close proximity to the fiberbot setup. To accurately align the sensor with respect to the fiberbot, a digital microscope (VHX-6000, Keyence, Japan) was used. Similar to the previous characterizations, the force measurements (at zero displacements) were acquired by performing a power sweep from 0.105 to 1.155 W with ≈105-mW increments. The input power was also maintained for each measurement for 1 minute, and the average contact force for the last 30 s was calculated. The same experiment was repeated five times, and the mean force and its SD for each input power were calculated.

Additional characterization experiments were performed to further assess the performance of the proposed system by making use of additional structures/devices and setup rearrangements. One such experiment consisted of exploring the relationship between the length of the actuated fiberbot and its corresponding tip displacement. A clamp was used to tighten the fiberbot at different points, thereby ensuring that only part of the fiberbot could move freely during actuation. The same triangulation laser sensor was used to measure the displacement of the fiberbot tip in the horizontal direction. As demonstrated in Fig. 3E, a power sweep from 0 to 1.449 W with ≈161-mW increments and intermitted by approximately 2.5 min was performed to capture the effect of the free length of the fiberbot on its tip displacement.

The thermal images shown in Fig. 3 (F and G) were captured using a highly sensitive medium-wave infrared (MWIR) camera (FLIR X6900sc MWIR, Teledyne FLIR LLC, USA) to (i) visualize the temperature distribution profile along the fiberbot’s length, (ii) visualize the effect of different fiberbot lengths on its displacement, and (iii) compute the temperature gradient between the opposing sides of the fiberbot (actuated side and passive side) as a function of the applied power for horizontal displacements.

Using the power measurement circuit (fig. S5B), we characterized the change in resistance of the embedded wires due to fiberbot actuation by applying 10-V input steps, as shown in Fig. 3H. The displacement of the fiberbot tip was also acquired using the same laser displacement sensor. The velocity profiles presented in fig. S7 were obtained by (i) applying different voltage inputs, (ii) measuring the displacements using the displacement sensor for each input voltage, (iii) polynomial curve fitting of the measured displacement, and (iv) calculating the derivative of the curve fitted displacement with respect to time to get the approximate velocity profile of the fiberbot. Last, the hysteresis plot in Fig. 3I was obtained by performing an input power sweep from 0 to 1 W and vice versa in 125-mW incremental steps.

### Open- and closed-loop control mechanisms for fiberbot actuation

In the initial tests, an open-loop control mechanism was implemented to actuate the fiberbot to follow a specific path. By using the measured power versus displacement curves, it was assumed that the power applied through a pair of stainless-steel wires is proportional to the displacement of the fiberbot tip as given below

$$A=\beta P=\beta ∙\frac{V^{2}}{R_{e}}=ϒ\Delta T$$ (1)

where *A* represents the amplitude displacement, *P* is the applied power, β is a constant derived from the power-displacement calibration, Δ*T* is the temperature difference between the opposing sides of the fiberbot, and ϒ is a constant derived from the temperature-displacement calibration.

The position of the fiberbot tip is represented by a 2D coordinate system, with the origin defined as the equilibrium position of the fiberbot (before actuation). Different paths (raster, square, star, spiral, and circle) were generated in MATLAB (version 2020, MathWorks Inc., USA) as an ordered set of [*X* and *Y*] input coordinates to the open-loop controller. The flowchart shown in fig. S8 describes the methodology behind the open-loop controller developed in LabVIEW. Using the set of input coordinates, the first task of the controller is to find the region for the initial [*X* and *Y*] coordinates from a set of eight regions spanning the 2D coordinate system. In vertical and horizontal displacements, only one pair of wires is powered based on the voltage calculated in Eq. 1. For diagonal displacements, two pairs of wires are powered on the basis of the electric power ratio between the pairs controlling the direction of inclination. Following the first point, the remaining points of the path are executed one by one using the aforementioned approach to determine the next pair(s) of wire(s) to be driven and the corresponding voltage(s). However, the results obtained by this open-loop controller have shown significant deviations from the ideal path (reference) due to the inherent slow thermal response of electrothermal actuation and nonlinearities such as the effect of gravity affecting the horizontal and vertical motions differentially. To compensate for these positional errors, the input paths were manually and iteratively optimized.

For the closed-loop control strategies illustrated in fig. S10, four proportional-integral derivative (PID) visual instruments (VIs) (version 2018, National Instruments, USA) were used to control the four pairs of wires. Here, we used the opposing pairs of wires to control one dimension of the overall 2D motion. For example, to move the fiberbot’s tip to the left-up direction, the input voltages across the left and top pairs of wires are decreased; concurrently, the input voltages across the right and bottom pairs of wires are increased. In the case of optical-based feedback, the tracked position for the fiberbot’s tip (process variable) was deducted from the input position (desired set point), and thus the deviation was calculated. On the basis of the calculated deviation, the manually tuned PID controller and transfer function (mapping displacement to voltage; Eq. 1) of the fiberbot determine the voltage input for wires.

For temperature-based feedback, we mapped the desired fiberbot tip displacement to the corresponding temperature difference between the opposing sides of the fiberbot (Eq. 1). Then, the temperature difference measured (process variable) using the thermocouples (406-590, TC Direct, UK) was deducted from the input temperature difference (desired set point) to calculate the deviation. To remove noise from the temperature signals obtained by the thermocouples, we implemented low-pass filters, which effectively smooth out these signals before feeding them to the controller. Using the computed deviation, the manually tuned PID controller and transfer function (mapping temperature difference to voltage; Eq. 1) of the fiberbot calculate the voltage input for the wire pairs.

### Integration with the surgical laser and the REIMS platform

A Xevo G2-XS quadrupole time-of-flight (Waters, MA, USA) equipped with a prototype REIMS source was used for the experiments. A CO_2_ laser source (OmniGuide, USA) was coupled with a flexible hollow-core PBG fiber (0.9 mm in OD; fiber was stripped of its polymeric jacket; OmniGuide, USA) to guide CO_2_ (wavelength = 10.6 μm). The PBG fiber was subsequently inserted into the central channel of the fiberbot to perform simultaneous scanning and ablation of ex vivo tissue samples. The ensemble of these different components leads to the realization of an intelligent knife (iKnife) system for intra-operative diagnostic mapping (*54*, *78*).

The fiberbot was placed in a bespoke enclosure to create a well-controlled and stable environment. The enclosure was designed using a 3D CAD software (SolidWorks, Dassault Systèmes, France) to accommodate the fiberbot, its electronic circuitry, and an ex vivo tissue slide holder. This enclosure and the tissue slide holder are 3D printed in VeroClear material using a PolyJet printer (Objet500 Connex, Stratasys Ltd., USA). The holder was designed to be moved and mounted onto the shell’s base at different positions (i.e., adjustable) to accommodate different lengths of fibers. The tissue samples were ablated by moving the embedded laser fiber along a preoptimized path of parallel lines.

A LabVIEW VI (version 2018, National Instruments, USA) was developed to (i) actuate the fiberbot, (ii) switch on and off the laser source for ablation (“on” for the parallel lines part of the path, “off” for the intermediate motions between the lines), and (iii) trigger the mass spectrometer to start the analysis of the aerosol. The mass spectral data were acquired between 50 and 1200 mass/charge ratio (*m*/*z*) in the negative ion mode at the rate of 5 scans/s.

### Mass spectral data processing and heatmap generation

The mass spectral data were acquired using MassLynx (version 4.1, Waters, USA) and processed using Abstract Model Builder (version 1.0.2059.0., Waters Research Center, Hungary). First, the data were processed using data binning between 50 and 1200 *m*/*z*, with a bin width of 0.1 *m*/*z*. The data were then imported into a custom-made script, where the mass spectral data acquired while ablating the tissue (i.e., laser on) were selected to generate the parallel line’s heatmap. The time intervals as to when the laser was activated and deactivated were obtained from a data file generated by a dedicated LabVIEW VI that keeps a time-stamped log of the main control parameters. Next, the selected data were plotted in a 2D heatmap with equal pixel sizes for the 200 highest mass bins.

Having equally sized pixels introduces inaccuracies to the heatmap generated due to the change in velocity of the fiberbot as it moves along the parallel line path. This change in velocity (i.e., acceleration at the beginning and deceleration at the end of each line) stems from the relatively slow thermal response of electrothermal actuation. Because the mass spectral data are acquired at a fixed sample rate (5 scans/s), and the distance traveled during the acquisition of each scan is variable, there is a need to correct the size of each pixel based on the actual distances traveled by the fiberbot. These distances were obtained by processing the images of a video recording of the fiberbot in motion using MATLAB (version 2021, MathWorks, USA). A more accurate heatmap was then generated by adjusting the pixel sizes based on the corrected distances at each mass spectral sampling position. The resulting pixel images were overlaid onto the optical image of the post-ablated ex vivo tissue slide.

### pCLE system integration

Figure S15 shows the schematics of the pCLE system, with the full description of the system provided in (*79*)*.* For this work, the illumination consists of a compact laser diode delivering light at 488 nm, and a high-resolution flexible AQ-Flex19 fiber bundle probe from Cellvizio (0.91 mm in diameter, 325-μm FOV, 3.5-μm resolution; Mauna Kea Technologies, France) was used for scanning. A sterile plastic sheath with ~50-μm thickness was kept between the probe’s tip and tissue surface to avoid contamination. During scanning, a time-averaged power of ~1.6 mW is delivered to the tip of the probe. For the complementary metal-oxide semiconductor camera, an exposure of 30 μs, which corresponds to a slit width of about 2.6 μm, was chosen.

### pCLE data acquisition and processing protocol

In terms of the preparation of the urology specimens, small cut-outs from freshly excised urology specimens were acquired from the histopathology laboratory in Charing Cross Hospital (London, UK) and snap-frozen at −80°C. These specimens were obtained from consented patients using Imperial College London Tissue Bank’s (ICHTB’s) ethical protocol following the R-19016 project, with ICHTB Human Tissue Authority license number 12275 and Research Ethics Committee (REC) approval reference 17/WA/0161. The neoplastic tissue was obtained from macroscopically visible tumor sites. Before image acquisition by the proposed pCLE fiberbot-actuated system, the frozen cut-outs were thawed at room temperature for 5 min. Following this, the samples were immersed in a 0.1% concentration of acriflavine hydrochloride solution for 30 s to allow the tissues to be stained, followed by rinsing with water to remove any excess agent. The flexible fiber bundle probe was autonomously steered at its distal end along a preoptimized spiral path onto the tissue surface, and images were obtained in real time.

The pCLE images were acquired and recorded at 120 frames/s. Endomicroscopy video loops were recorded for about 60 s from four to six sites on the tissue surface. The data were post-processed to remove the fiber bundle pixelation artifacts (caused by the fiber cores) by convolution with a 2D Gaussian filter (sigma = 1.6 pixels, 1.4 μm on the bundle). This was followed by mosaicking the video frames (i.e., stitching overlapping frames) to produce images with a larger FOV while still maintaining microscopic resolution that could be comparable to histology slides. A fast normalized cross-correlation (NCC) algorithm was used for pair-wise image registration (*80*)*.* The primary intent behind using the fast NCC was to use fast fourier transform to evaluate, in one pass, the correlation coefficient between consecutive image frames *I_k_* and *I*_*k*+1_. A 2D correlation map was generated using

$$C_{\text{NCC}}\left(u,v\right)=\frac{\sum [I_{k}\left(x,y\right)-I_{k}][I_{k+1}\left(x-u,y-v\right)-I_{k+1}]}{\sum {\left[I_{k}\left(x,y\right)-I_{k}\right]}^{2}+\sum {\left[I_{k+1}\left(x-u,y-v\right)-I_{k+1}\right]}^{2}}$$ (2)

where *I_k_* is the average pixel value of the image *K*; *x*, *y* are pixel coordinates; and *u*, *v* is the translational shift. The translational shift was obtained from the location of the maximum correlation peak in the 2D correlation map, given by

$$\hat{C}(I_{k},I_{k+1})=\text{arg}\text{max}\left[C_{\text{NCC}}\left(u,v\right)\right]$$ (3)

As the final step, a weighted-average feathering function for image blending was implemented to reconstruct an output mosaic with minimum visible seams.

### In vivo animal testing setup

A female Yorkshire swine of 45 kg was used for the animal trial. All procedures were conducted in accordance with the protocol approved by the Acibadem Ali Aydinlar University Animal Experiment Local Ethics Committee. The trial commenced by premedicating the swine with a combination of ketamine (preanesthetic and analgesic drug) and xylazine (sedative and analgesic drug with some muscle relaxation properties). After inducing anesthesia using propofol (anesthetic drug) through an intravenous cannula (inserted into the swine’s left ear vein), the swine was endotracheally intubated. During the trial, isoflurane (anesthetic drug) was used to maintain anesthesia, and metamizole (pain killer) to treat discomfort. Using the anesthesia machine (Leon Plus, Löwenstein Medical SE & Co. KG, Germany), the swine’s ventilation was maintained using mechanical ventilation under the continuous monitoring of its physiological status. At the end of this study, to facilitate euthanasia, the isoflurane dose was increased to a maximum (5%), and potassium chloride was administered intravenously after a 5-min interval.

### Histopathological assessment

The specimens from the liver and spleen were placed in an aqueous solution of formaldehyde and sent to an independent laboratory (Acibadem pathology/Nisantasi Pathology Group, Istanbul) for histopathological assessment. First, the tissue specimens were macroscopically examined. Subsequently, the tissue samples were serially sectioned, and all tissue planes were mapped and sampled. After embedding the tissue samples with paraffin, the samples were cut into 4-μm-thick slices and stained with hematoxylin and eosin (H&E). The H&E slides were then assessed by an expert pathologist to investigate if there were any signs of thermal injury.

## Supplementary Materials

### This PDF file includes:

Notes S1 to S9 Figs. S1 to S17 Tables S1 to S5 Legends for movies S1 to S7 References
