# Supplementary material for: Fiberbots: Robotic fibers for high-precision minimally invasive surgery
Source: Sci Adv. 2024 Jan 19;10(3):eadj1984. doi: 10.1126/sciadv.adj1984 (PMC10798568; doi:10.1126/sciadv.adj1984)
Supplement: Supplementary file 1 — Notes S1 to S9 Figs. S1 to S17 Tables S1 to S5 Legends for movies S1 to S7 References [file sciadv.adj1984_sm.pdf]

Science Advances
AAAS

Supplementary Materials for

Fiberbots: Robotic fibers for high-precision minimally invasive surgery

Mohamed E. M. K. Abdelaziz *et al.*

Corresponding author: Burak Temelkuran, b.temelkuran@imperial.ac.uk

Sci. Adv. **10**, eadj1984 (2024)
DOI: 10.1126/sciadv.adj1984

The PDF file includes:

Notes S1 to S9
Figs. S1 to S17
Tables S1 to S5
Legends for movies S1 to S7
References

Other Supplementary Material for this manuscript includes the following:

Movies S1 to S7

Supplementary Text

Supplementary Note S1: Numerical simulations of the fiberbots

To build upon the analytical model and further explore the temperature distribution and motion behaviour of the fiberbot, we simulated the actuation principle by designing a three-dimensional (3D) model of the fiber embedded with stainless-steel wires using a finite element analysis (FEA) software (ANSYS R18.2, ANSYS Corp., USA). The finite element modules, including Thermal-Electric, Transient Thermal, and Static Structure analysis, were used to: (a) examine the effect of thermal expansion on the polymer-metal composite, and (b) assess different fiber cross-sectional structures.

We investigated a solid polymer fiber body embedded with four pairs of wires and a central channel. The length of the fiber is 10 cm. In all the simulations below, we fixed the device's inner diameter to 1mm, which was determined by the diameters of the smallest pCLE and PBG fibers available in the market (i.e., roughly 0.9 mm in diameter). An air domain (specific heat = 1003 J/(kg°C)) was defined inside the central channel of the fiber to simulate the real-case scenario.

In the simulation environment, we employed the sweep mesh method to generate the 3D computational mesh for the fiber structure. The sweep mesh method was used to maintain high solver accuracy while reducing the mesh cell elements, leading to quicker solve times. The 3D fiber model was divided into 20 elements in the axial direction, and triangular elements with an edge resolution of 0.04 mm were used to mesh the cross-section. For the Thermal-Electric analysis module, we applied a voltage potential difference across two resistance wires and obtained the Joule heat generated rate. The power generated by Joule heating was then imported into the Transient Thermal module and used to set the wires as a heat source at the beginning of the simulation time. The convection heat transfer coefficient (2.5 x 10^{-5} W/(mm^2·°C)) and emissivity (0.92) were set at the outer surface of the fiber. The ambient temperature was set at 25 °C.

From the Transient Thermal analysis, we obtained the temperature distribution of the fiber. The temperature of the encapsulating polymer and the stainless-steel wires were imported into the Static Structure analysis module to calculate the deflection of the fiber. Under this module, the fiber model was fixed at one end, while the other end was allowed to deform freely in space (resembling a cantilever structure), with no-slip boundary conditions imposed at the interface between the metal and polymer. By actuating the pair of wires, one-directional deformation of the fiber was achieved.

Supplementary Note S2: Fiber robot structural optimization and material selection

To explore the effect of wall thickness on the performance of fiberbot. We performed a parametric sweep of the outer diameter from 1.5 mm to 2.0 mm at 100 µm intervals (i.e., 50 µm wall thickness intervals). In this experiment, the fiber body was initially defined as PC (density = 1.2 g/cm^3, coefficient of thermal expansion = 2.1 x 10^{-5} °C^{-1}, Young's modulus = 2 x 10^9 Pa, Poisson's ratio = 0.42, isotropic thermal conductivity = 0.22 W/(m.°C), specific heat = 1200 J/(kg. °C)), whereas the wires were defined as stainless-steel (density = 7.5 g/cm^3, coefficient of thermal expansion = 1.04 x 10^{-5} °C^{-1}, Young's modulus = 1.9 x 10^{11} Pa, Poisson's ratio = 0.265, isotropic thermal conductivity = 25 W/(m.°C), specific heat = 490 J/(kg.°C), isotropic resistivity = 6.9 x 10^{-7}

Ω.m). The input power is fixed to 1 W. The results are shown in Fig. S1, and details are listed in Table S3. The results of the parametric study suggest that the effect of wall thickness is minimal, with a 0.91 mm increase (30.4% increment) in tip displacement, for a 50% decrease in wall thickness (i.e., from 0.5 mm to 0.25 mm). Similarly, the maximum temperature increases by approximately 12.4°C (30% increment) for the 50% decrease in wall thickness.

To explore the effect of wire positioning inside the polymer material, another parametric sweep was performed with the wire positions changing from 0.60 mm to 0.90 mm relative to the fiber's central axis (denoted by Owp) at 50 µm intervals. For this set of simulations, we fixed the input power to 1 W and set the inner and outer diameters to 1 mm and 2 mm, respectively (see Fig. S2 and Table S4). In terms of displacement, the closer the wires are to the fiber's central axis, the larger the fiber's tip displacement (0.38 mm increase). Temperature-wise, by placing the wires away from the fiber's outer surface, the hot (actuated) side's surface temperature decreases by 6.2°C. As demonstrated here, we can also conclude that the contribution of the different wire positions to the tip's displacement is minimal.

To demonstrate the effect of using different thermoplastic polymers, we performed additional simulations to demonstrate the insignificant effect of Young's modulus on the tip deflection. For this set of simulations, we employed polymers that were used by other research groups to create 3D printed preforms. The average value for the mechanical and thermal properties employed is listed in Table S5. The input power is again fixed to 1 W and the device's inner and outer diameter are fixed to 1 mm and 1.65 mm, respectively. As presented in Table S5, most of the 3D printed polymers are amorphous in nature and have a very similar modulus to that of polycarbonate (PC). Note: amorphous polymers are usually favorable for fiber drawing because their viscosity varies quasi-continuously between the solid and liquid states, in contrast to crystalline material. This ensures that the preform can be drawn at reasonable speeds with self-maintaining structural regularity. From the results, the differences in tip displacement of the five polymers are insignificant.

After determining the structure and materials through the above simulations, we performed a set of simulations with similar parameters to the real fiberbots (Fig. S3, A to E). The thickness of the fiber is set at 1.65 mm with a length of 10 cm. The fiber body is defined as PC. Transient Thermal analysis results in Fig. S3B show the resulting temperature distribution in the presence of a central channel. The steady-state temperature difference between the actuated and passive sides of the fiberbot was 21.6°C (Fig. S3C), with the Static Structure analysis resulting in a tip displacement of 1.197 mm (Fig. S3E).

Supplementary Note S3: Electronic circuitry for fiber actuation and sensing

The fiber was actuated using an electronic circuit designed with four independent voltage channels, which impose an electrical current "i" to carry out the fiberbot motion in four directions (X-, X+, Y- and Y+). This configuration was realized using two voltage-to-current converters built around power amplifiers (OPA548, Texas Instruments, USA) mounted in a non-inverting topology. By imposing a voltage difference at the output of each amplifier (V_0^{out} and V_1^{out}, respectively), a current flow that is inversely proportional to the resistance of the wire conduction path (< 100 Ω) is generated through a single pair of wires running on the same side of the fiber. Twisting and

connecting the distal ends of the pair of wires guaranteed a closed loop for current circulation inside the fiberbot. By considering the voltage signals (V_0^{in} and V_1^{in}) produced by the two channels inside a digital-to-analog (DAC) conversion board (NI-9264 DAC, National Instruments, USA) docked into a real-time controller (CompactRIO cRIO-9025, National Instruments, USA), and the equivalent resistance value for the respective wire-pair (R_e), the current can be calculated as follows:

$$i = \frac{(V_0^{out} - V_1^{out})}{R_e} = \frac{2(V_0^{in} - V_1^{in})}{R_w + R_w + R_S} \approx \frac{(V_0^{in} - V_1^{in})}{R_w} \quad \text{(SI-1)}$$

where R_S is considered close to 0 Ω due to the high conductivity of the silver adhesive glue (CW2205, Chemtronics, USA) employed to bond each pair of wires, whereas R_w is assumed similar between the pair of wires. Irrespective of the direction of current flow, the electric power dissipated by one pair of wires is equivalent to the quadratic factor presented in Equation SI-2.

$$P_{electric} = i^2 R_e = 2R_w \frac{(V_0^{in} - V_1^{in})^2}{R_w^2} = 2\frac{(V_0^{in} - V_1^{in})^2}{R_w} \quad \text{(SI-2)}$$

An additional relay (G6K-2P-Y, Omron, Japan) activated by an emitter-follower transistor circuit topology (2N2222, Multicomp Pro, USA) was intercalated between each current loop to form an automatic switch that disconnects the fiberbot from the electronics in the event of an emergency.

The above circuit was converted into a printed circuit board (PCB) using EAGLE software (Autodesk, USA) as part of a larger electronic assembly that also includes the PCBs for power and temperature feedback measurements, as shown in Fig. S5. The bottom PCB in the assembly holds the electronic circuit responsible for driving 8 voltage-to-current converters (channels), whereas the middle PCB contains the proximal end of the electrothermal fiber with the internal wires soldered to exposed electrical pads on the surface of the board. Additional ultraviolet (UV)-curable adhesive (3525, Loctite, Germany) was poured above these soldering anchors to protect the wires and ensure mechanical stability to the fiber during actuation.

Power measurements were performed within the middle PCB by two electronic channels that measure, separately, the electric current flowing through one pair of wires and the voltage registered between the proximal ends (or input terminals) of each wire (Fig. S5B). Although a theoretical formula for the generated power was derived previously, in practice, some signal drifts, interferences, and changes in wire resistance during actuation can lead to fluctuations in power level. So, more accurate measurements were needed to better characterize the fiberbot performance in terms of electrical metrics. In order to achieve this, three amplifiers (OPA604, Texas Instruments, USA) in a buffer circuit topology were intercalated in sequence along the current loop formed by each pair of wires to produce two voltage measurements ($V_{P1} - V_{P2}$) and ($V_{P2} - V_{P3}$). Two amplifiers (Amp1 and Amp2) sense the voltage drop across a fixed-value resistor (10 Ω) in the loop, which enables the calculation of the magnitude for the circulating current as:

$$i = \frac{2(V_{P1} - V_{P2})}{10\ \Omega} \quad \text{(SI-3)}$$

Similarly, the voltage originated at the terminals of the pair of wires is detected by two set of amplifiers (Amp2 and Amp3) and calculated according to the equation below.

$$V = 2(V_{P2} - V_{P3}) \quad \text{(SI-4)}$$

The voltage measurements (V_{P1}, V_{P2}, V_{P3}) were acquired using an analog-to-digital (ADC) converter (NI-9205 ADC, National Instruments, USA) docked into another slot of the same real-time controller (cRIO-9025), with the measured power calculated according to Equation SI-5.

$$P_{meas} = i \cdot V = \frac{4(V_{P1} - V_{P2})(V_{P2} - V_{P3})}{10\ \Omega} \quad \text{(SI-5)}$$

Furthermore, the resistance of a single wire pair was also estimated using Equation SI-6, thus, allowing real-time feedback measurements for both power and resistance during fiberbot actuation.

$$R_e = \frac{P_{meas}}{i^2} = \frac{(V_{P2} - V_{P3})}{(V_{P1} - V_{P2})} \cdot 10\Omega \quad \text{(SI-6)}$$

In terms of temperature feedback measurements, four K-type thermocouples (406-590, TC Direct, UK) were placed along the outer circumference of the fiber next to the pair of wires, with 90° separation between them. Polyimide film tape (5413, 3M, USA) was used to hold the thermocouples at the measurement junction. Each thermocouple was then connected to an instrumentation amplifier (AD8497, Analog Devices, USA) at the reference junction to detect the voltage levels induced by the temperature variations (5 mV/^{o}C), as shown in Fig. S5D. The voltage measurements (V_T) were acquired using the same ADC converter (NI-9205 ADC), with the temperature value calculated by Equation SI-7.

$$T = \frac{V_T}{(5\ \text{mV}/^o\text{C})} \quad \text{(SI-7)}$$

Supplementary Note S4: Outer surface characterization

As the surface temperatures of the actuated fiber are above the biological safe limit of 43°C (Fig. 2G), we introduced active cooling by injecting compressed air through the central channel of the fiberbot. We placed the fiber alongside the laser displacement sensor (LK-G5000, Keyence, Japan) inside an incubating mini-shaker (VWR® Incubating Mini Shaker, VWR, UK) (Fig. S6) to set the environment temperature of the fiber to 38°C ± 1°C. A 21-gauge needle (SA7524, Adhesive Dispensing Ltd, UK) was connected from its hub to a 465 cm-long air hose assembly (350 cm-long polyethylene tubing (126-3103, RS, UK) connected to a 115 cm-long flexible PVC tubing (WZ-30526-18, Cole-Parmer, USA)). The shaft of the needle, on the other hand, was inserted into the central channel of the fiber. Two K-type thermocouples (406-590, TC Direct, UK) were placed alongside the outer circumference of the fiber next to the pair of wires being actuated in order to measure the outer surface temperature.

Our experiments have shown that, by using air at room temperature, we can decrease the surface temperature of the actuated side of the fiber from approximately 45.5°C down to 43°C. However, the temperature gradient between the opposing ends dropped from 24.2°C to approximately 9.2°C at 5 bar pressure, whereas the fiber tip's displacement decreased by 1.94 mm. Therefore, using a fiber with an outer diameter of 1.65 mm and 11 cm in length, the maximum tip displacement to avoid thermal tissue damage is 1.68 mm for an input power of 1 W (Fig. 3I).

Supplementary Note S5: Iterative compensation technique to optimize open-loop control

As shown in Fig. S8F, the measured path deviates from the desired path, with an average path error (i.e., the time-independent difference between the desired and measured paths) of 45.5 μm. This inaccuracy is primarily due to the actuator's large time constant, caused by the polymer's intrinsically low thermal conductivity. This delay caused the fiberbot to move toward the current command's desired position without reaching the previous desired position (target). Another factor contributing to the discrepancy is gravity, which becomes more significant with the decrease in Young's modulus of the polymeric material as temperature rises and the alignment of its molecules relaxes. These effects were compensated for by: (a) slowing down the speed with which new desired positions are reached in areas with sudden changes in motion direction; and (b) manually tuning the magnitude of the input voltages based on previous trials (Fig. S8, D to K). Using this iterative compensation technique, we decreased the average path error of a raster by 65.9% (Fig. 4D).

Supplementary Note S6: Electrical safety of the fiberbots

Most living tissues are capable of carrying electrical currents in their physiological state, which can induce harmful effects depending on the magnitude (> 100 μA), duration and regime of current propagation (i.e., direct current – DC, or alternating current, AC). Stimulation of body tissues with DC for extended periods of time has been shown to cause the most severe physiological impairments due to the interaction of direct conductive currents with tissues, leading to electrolysis and permanent migration of ionic species, resulting in tissue bruises and also burns from the generated DC heat (*81*).

To evaluate the electrical safety of our fiberbot, we conducted an experimental test to measure the level of current leakage from the tip of the fiber while actuated (i.e., current circulating through a pair of wires) and immersed inside a conductivity solution of 0.1413 S/m (HI7030L, Hanna Instruments), as shown in Fig. S12. The reason behind the selection of this conductivity level is related to the typical values recorded amongst biological tissues in the chest/abdomen cavity (namely, small intestine: 0.164 S/m, stomach: 0.164 S/m and lungs: 0.101 S/m (*82*)) by impedance spectroscopy methods within the α- and β-dispersion region bands of Schwan's dispersion theory (*81*, *83*). If electrical current escapes the fiberbot, it will travel through the solution due to the difference in electrical potential set between an immersed metal electrode and the electronic ground of the actuation device (acting as a current sink or lower impedance point), thereby creating a closed loop for current flow that can be detected by series intercalation of an ammeter (2100035, Farnell, UK). Due to proper electrical isolation at the tip of the fiberbot (achieved by recessing the internal resistive wires and application of cyanoacrylate), we were able to detect zero amperes of current from the ammeter (set in the μA range and resolution of 0.01 μA) for several input powers

in the range between 0.04 W and 2 W, thereby asserting the electrical safety of the proposed fiberbot during actuation, as observed also during the *in vivo* trial.

Supplementary Note S7: Temperature testing of fiberbot outer surface

To further validate the thermal safety of our actuated fiberbot, we conducted two experimental tests. In the first test, we manually held the actuated fiberbot (heated side at approximately 80℃) with our bare hand for a duration of two minutes, feeling a noticeable warmth without any discomfort. In the second test, we affixed two thermocouples independently—one to the actuated fiberbot and the other to a beef steak (Fig. S16). Initial temperature measurements recorded the fiberbot at 77.6℃ and the beef steak at 20.5℃. Subsequently, we attached the beef steak to the continuously actuated fiberbot for a duration of 30 seconds, simulating a real scenario of tissue-fiber attachment during surgical intervention. A substantial gradual decrease in the fiber's surface temperature from 77.6℃ to 34.2℃ was observed.

Supplementary Note S8: Fiberbot control user interface

The fiberbot's control interface is designed within the LabVIEW environment. Users can select the desired motion trajectory and adjust motion path dimensions via LabVIEW's front panel interface prior to the fiberbot's motion. Once the user clicks 'run' on the front panel, the control code is executed. Real-time analogue voltage readings from the four pairs of resistive wires can be conveniently monitored through the same panel.

Supplementary Note S9: Flexural rigidity calculation of thermally drawn polycarbonate fibers

We characterized the flexural rigidity of the thermally drawn PC fibers resorting to the characterization setup shown in Fig. S17. By using the Euler-Bernoulli equation, the maximum deflection of the catheter δ is defined as follows:

$$\delta = \frac{FL^3}{3EI}, \quad EI = \frac{FL^3}{3\delta} \qquad \text{(SI-8)}$$

where L is the length of the catheter shaft sample, and δ is the maximum deflection produced by the lateral force F exerted at the free end of the catheter shaft sample. The product of the modulus of elasticity of the catheter material (E) and the moment inertia of the cross-section of the catheter with respect to its neutral axis (I) are usually grouped and referred to as flexural rigidity (EI). Flexural rigidity is independent of the experimental setup, such that if other values for L and δ are chosen, the (EI) value remains the same. For the thermally drawn PC fibers in this study, the flexural rigidity was measured at 532.8 Nmm^2.

Figures

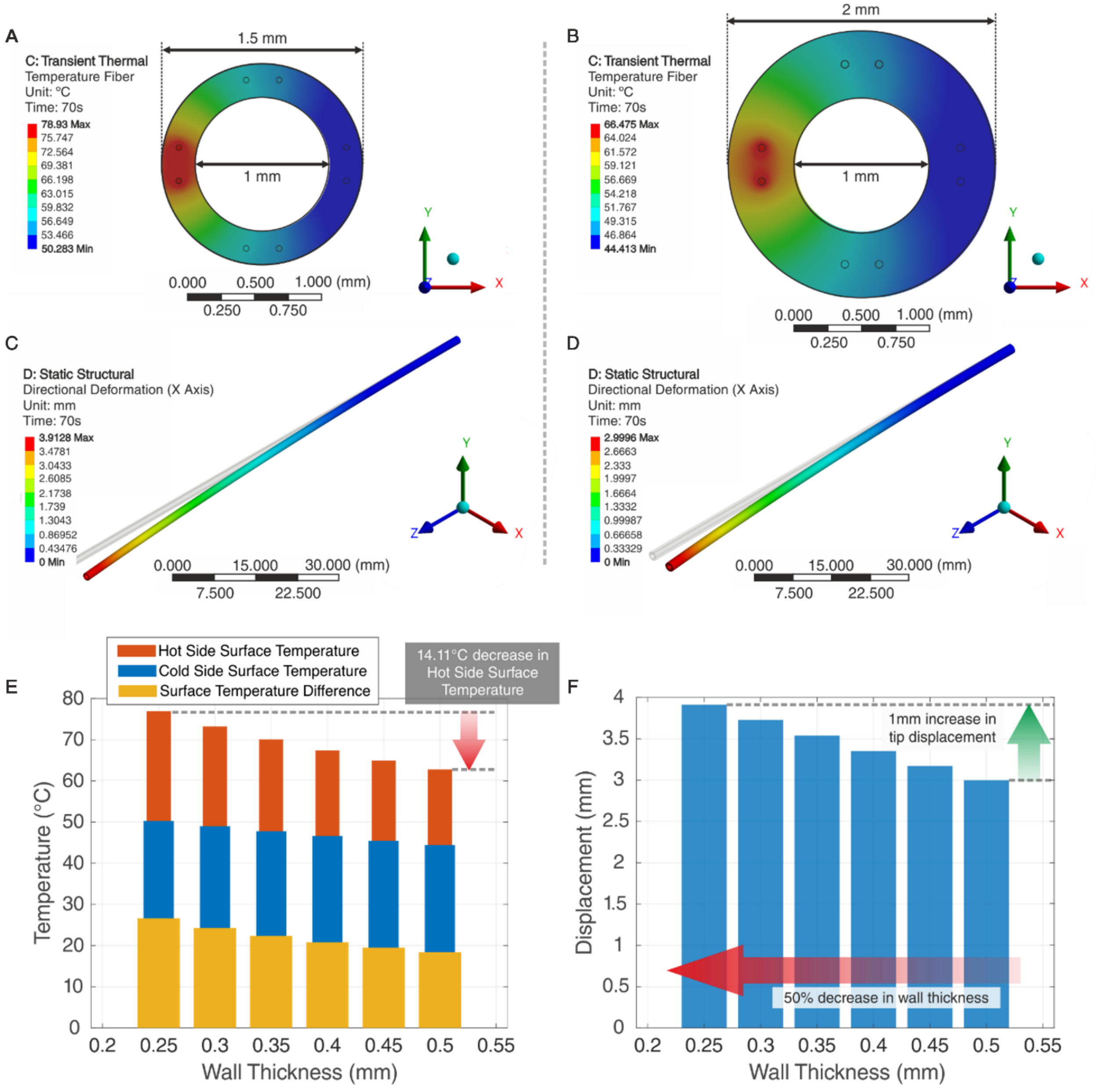

Fig. S1. Finite element analysis of the fiberbot (wall thickness). (**A, B**) Temperature distribution across the cross-section of the actuated electrothermal fiber with different wall thickness. (**C, D**) Directional deformation of the fiber with different wall thickness. (**E**) Temperature change (tip displacement) induced by the wall thickness effect. (**F**) Deformation change (tip displacement) induced by the wall thickness effect.

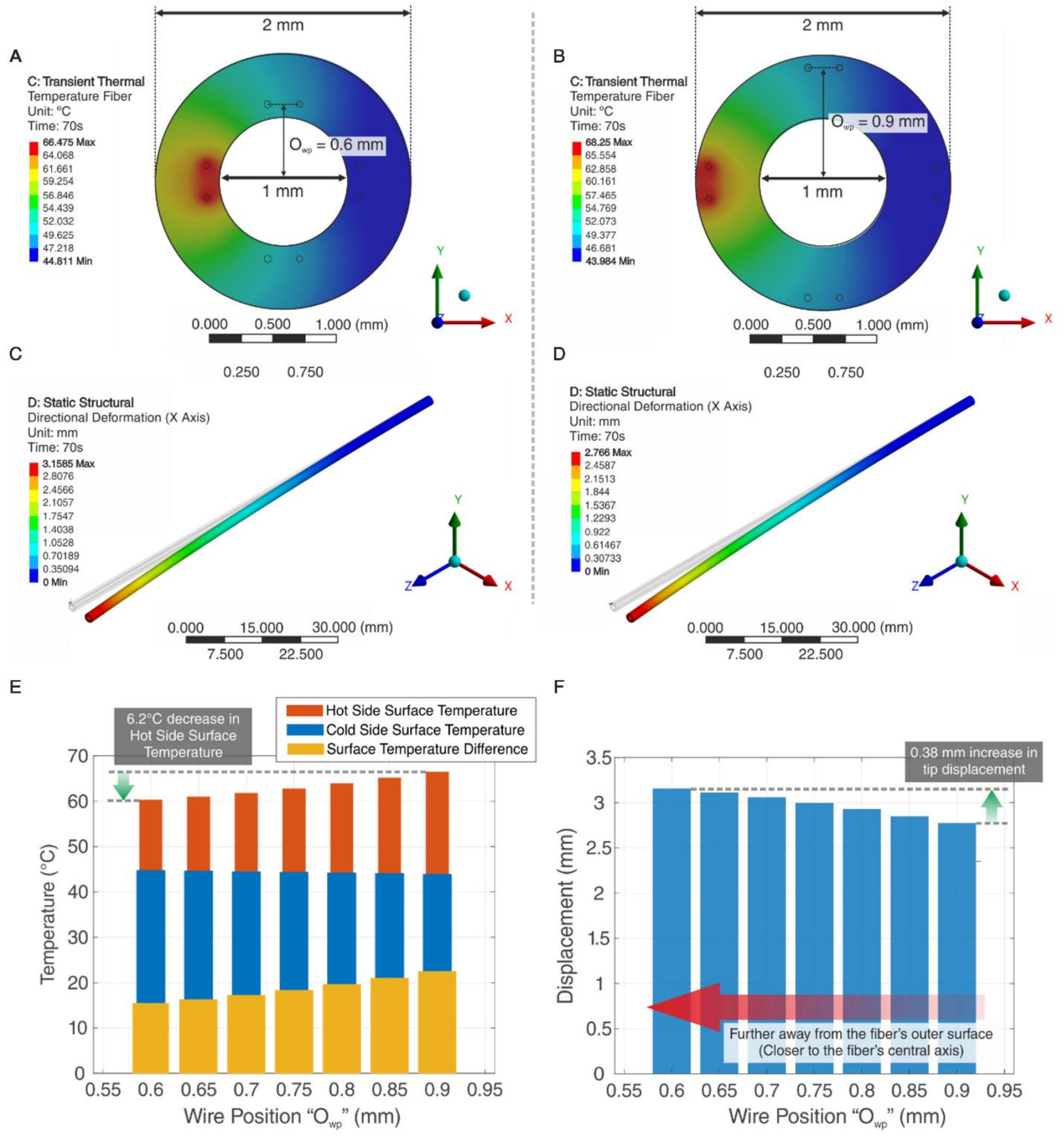

Fig. S2. Finite element analysis of the fiberbot (wire positioning). (**A, B**) Temperature distribution across the cross-section of the actuated fiber with different wire locations. (**C, D**) Directional deformation of the fiber with different wire locations. (**E**) Temperature change (tip displacement) induced by changing wire locations along the fiber wall. (**F**) Deformation change (tip displacement) induced by the same effect.

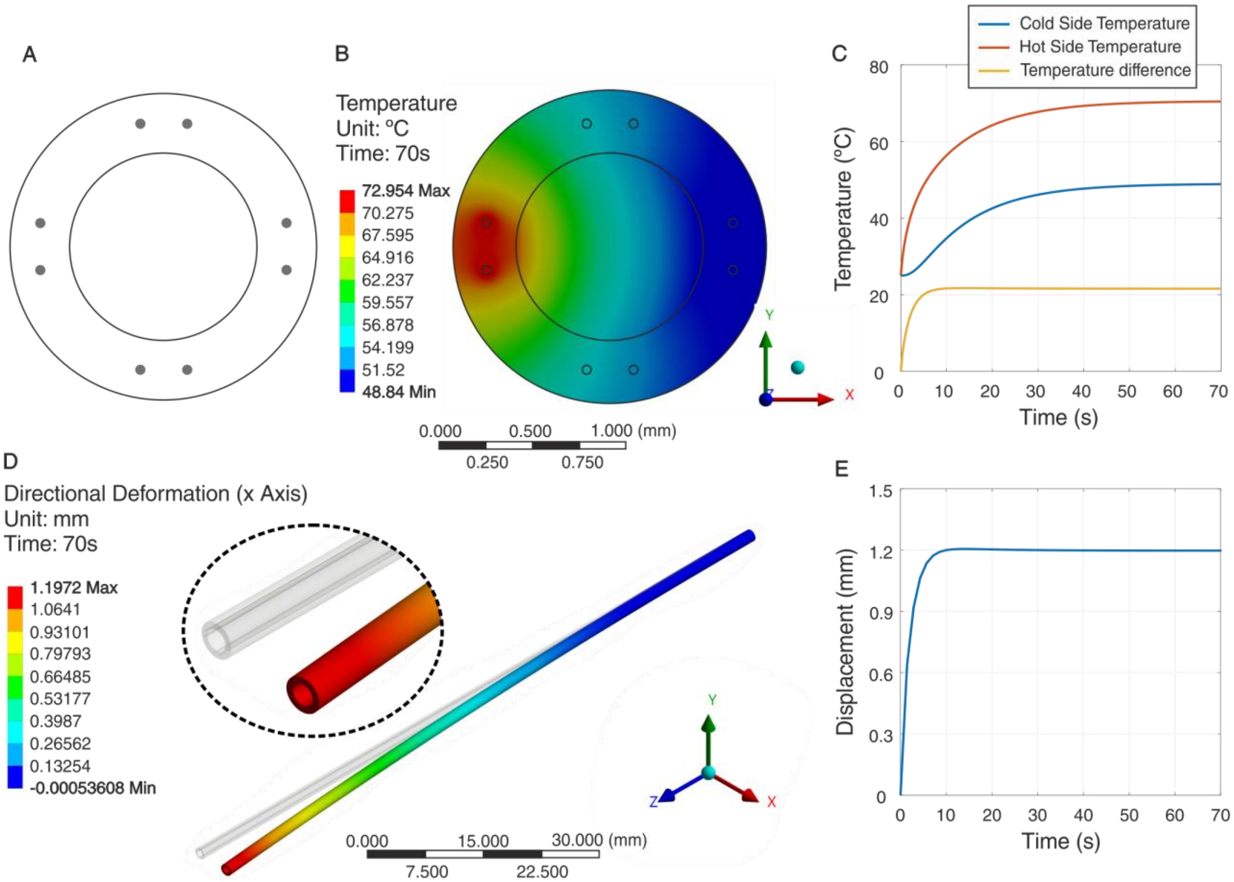

Fig. S3. Finite element analysis of the fiberbot (physical device). (**A**) Cross-section structure of fiberbot. (**B**) Temperature distribution across the cross-section of the actuated fiber. (**C**) Temperature changes on the fiber's actuated and passive sides over time, and the temperature difference between them. (**D**) Directional deformation of the fiber as seen from the side view (length of the fiber). (**E**) Deformation change (tip displacement) induced by the simulated electrothermal effect.

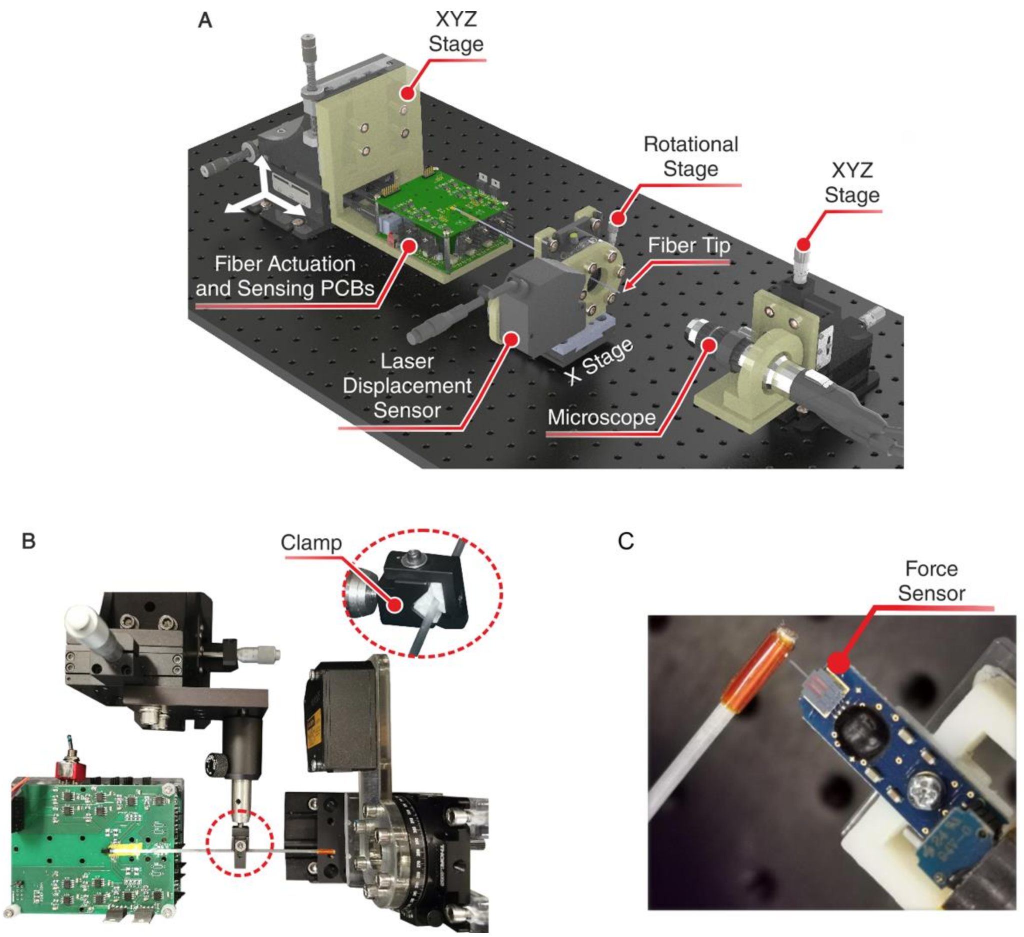

Fig. S4. Experimental setup used for characterization of the fiberbot motion. (**A**) Overall disposition of the different parts and equipment. (**B**) Setup used to characterize the effect of fiber length clamp on tip displacement (**C**) Commercial sensor used to sense the force applied by the fiberbot's tip.

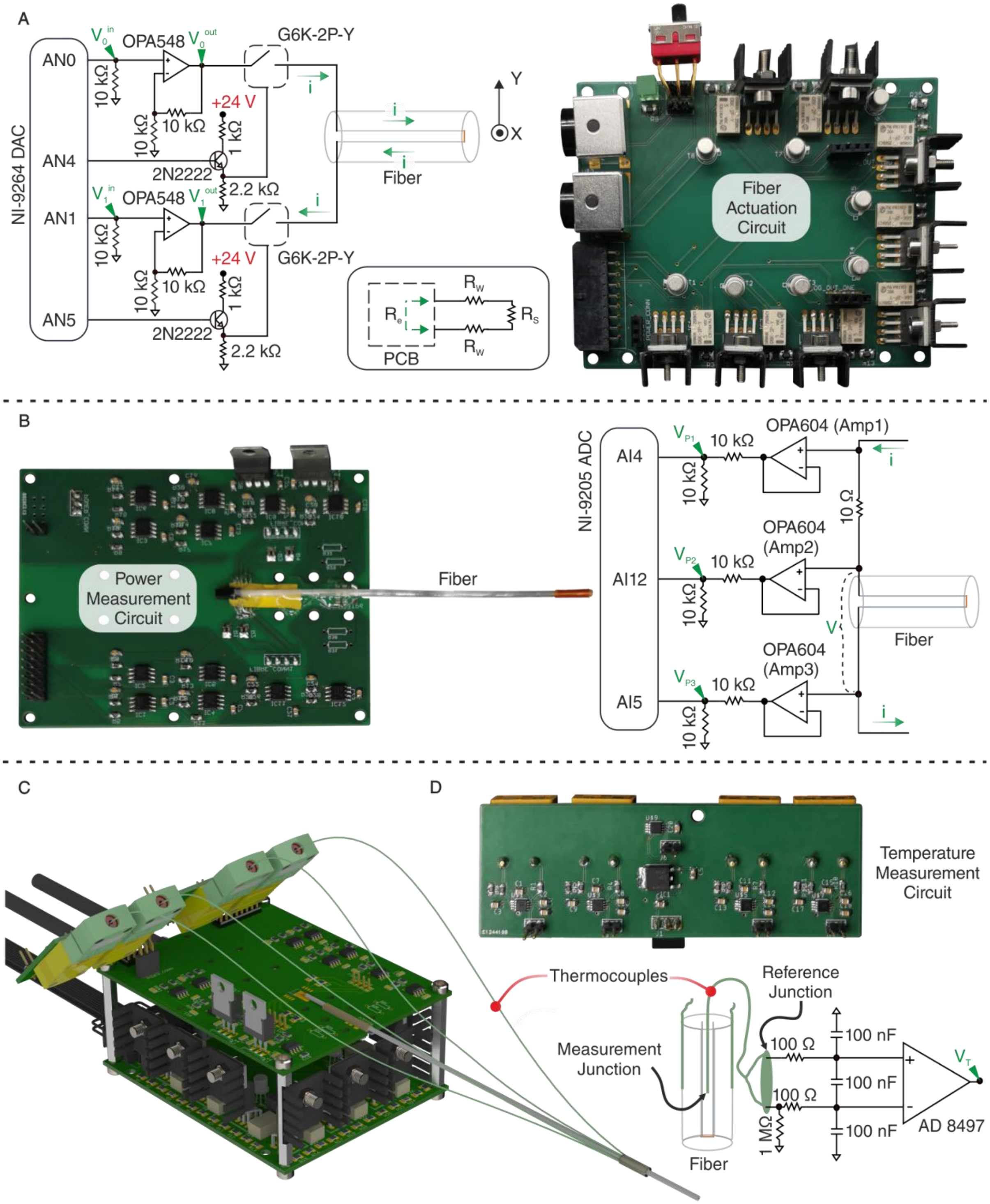

Fig. S5. Electronic circuitry for fiberbot actuation and sensing. (**A**) Simplified electronic schematic of the main components involved in generating the electrical current circulating through one wire-pair embedded inside the fiber (actuation). Inset: Equivalent resistance model of the wire pair. (**B**) Electronic schematic employed for real-time power feedback measurements. (**C**) Illustration of the assembled PCBs. (**D**) Electronic schematic employed to measure the temperatures utilizing external thermocouples placed around the outer surface of the fiber.

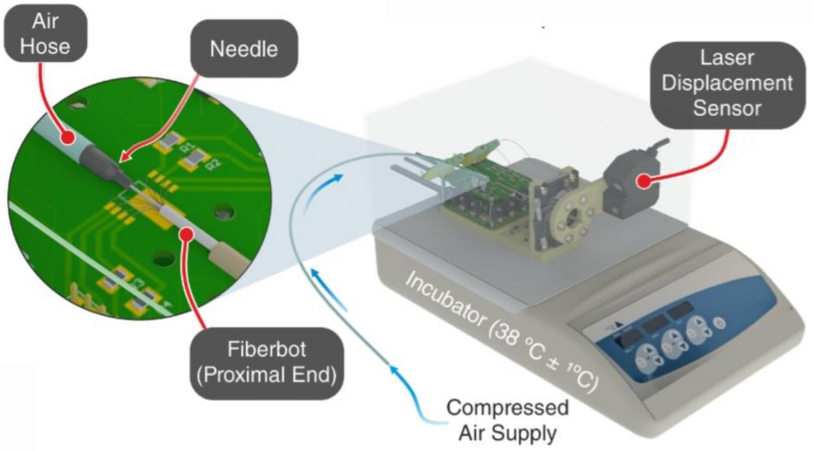

Fig. S6. Setup for demonstrating the ability to reduce the surface temperature of the fiberbot upon the application of compressed air through its central channel. Experiments conducted at 38°C ± 1°C. Inset: 21-gauge needle connected to an air hose and inserted into the central channel of the fiber.

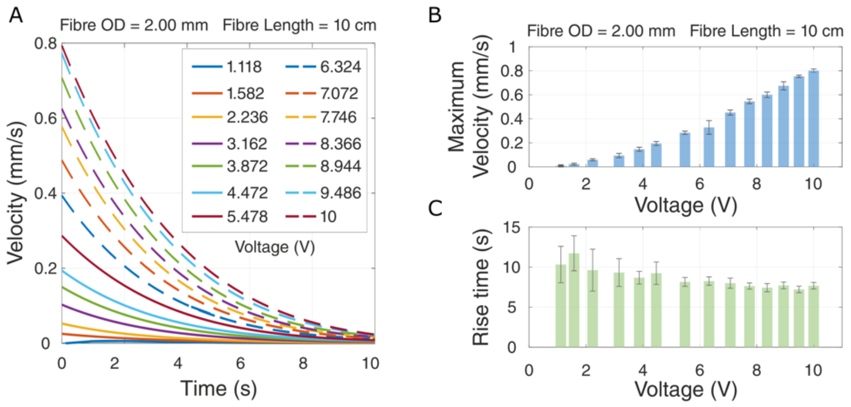

Fig. S7. Maximum velocity and rise time of the fiberbot. (**A**) Change in velocity of the fiber tip for different input voltage steps (starting from rest). (**B**) Fiber's maximum velocity. (**C**) Average time required by the fiberbot to move from 10% to 90% of its fully developed steady-state positions, i.e., rise time for the different input voltage steps.

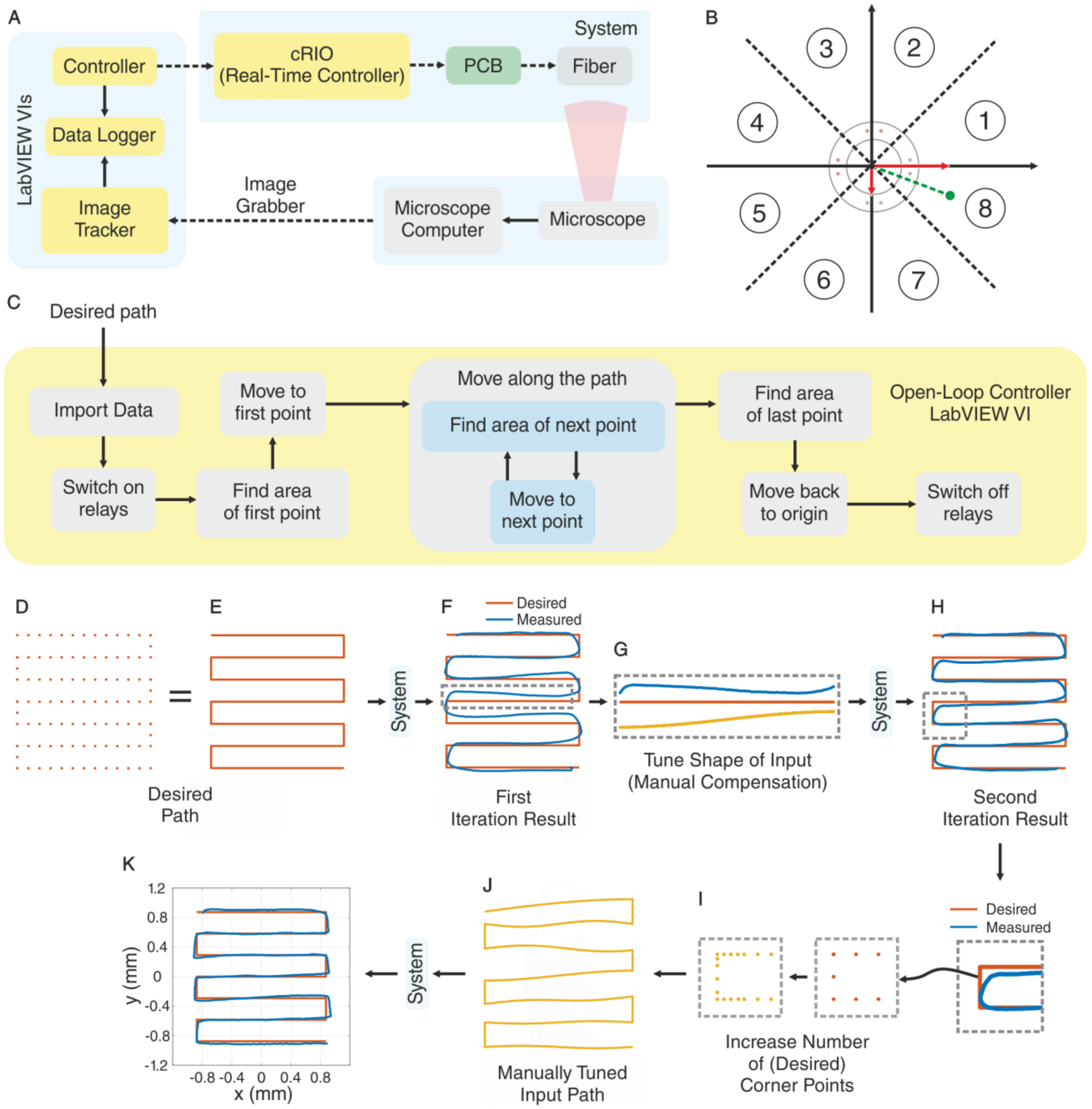

Fig. S8. Open-loop control mechanism for fiber actuation. (**A**) Schematic of the different modules, systems and graphical interfaces used to control the actuation mechanism with microscope image tracking of the fiber tip. (**B**) Cross-sectional plane spanned by the fiber tip divided into 8 regions for accurate steerability along the different projection axes. (**C**) Flowchart of the implemented open-loop controller. (**D**), (**E**) Desired trajectory path (raster) formed by input coordinate points (2D) to the controller. (**F**) – (**H**) Positional differences between the desired and measured (image) paths observed after the first iteration of the open-loop controller, highlighting the shift in the horizontal line shape necessary for manual tuning of the input trajectory (second iteration). (**I**), (**J**) Additional tuning of the trajectory by manual increase in the number of coordinate points allocated to the trajectory corners. (**K**) Planar overlap of the desired and measured paths after manual optimization with the open-loop controller.

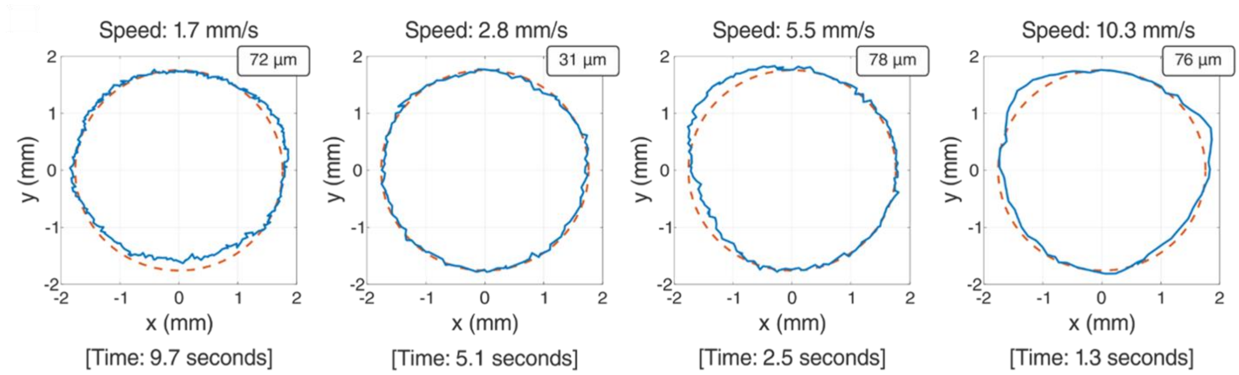

Fig. S9. Fiberbot commanded to follow a circular path at different speeds. The maximum achievable speed when the average errors are restricted to 80 µm is 10.3 mm/s.

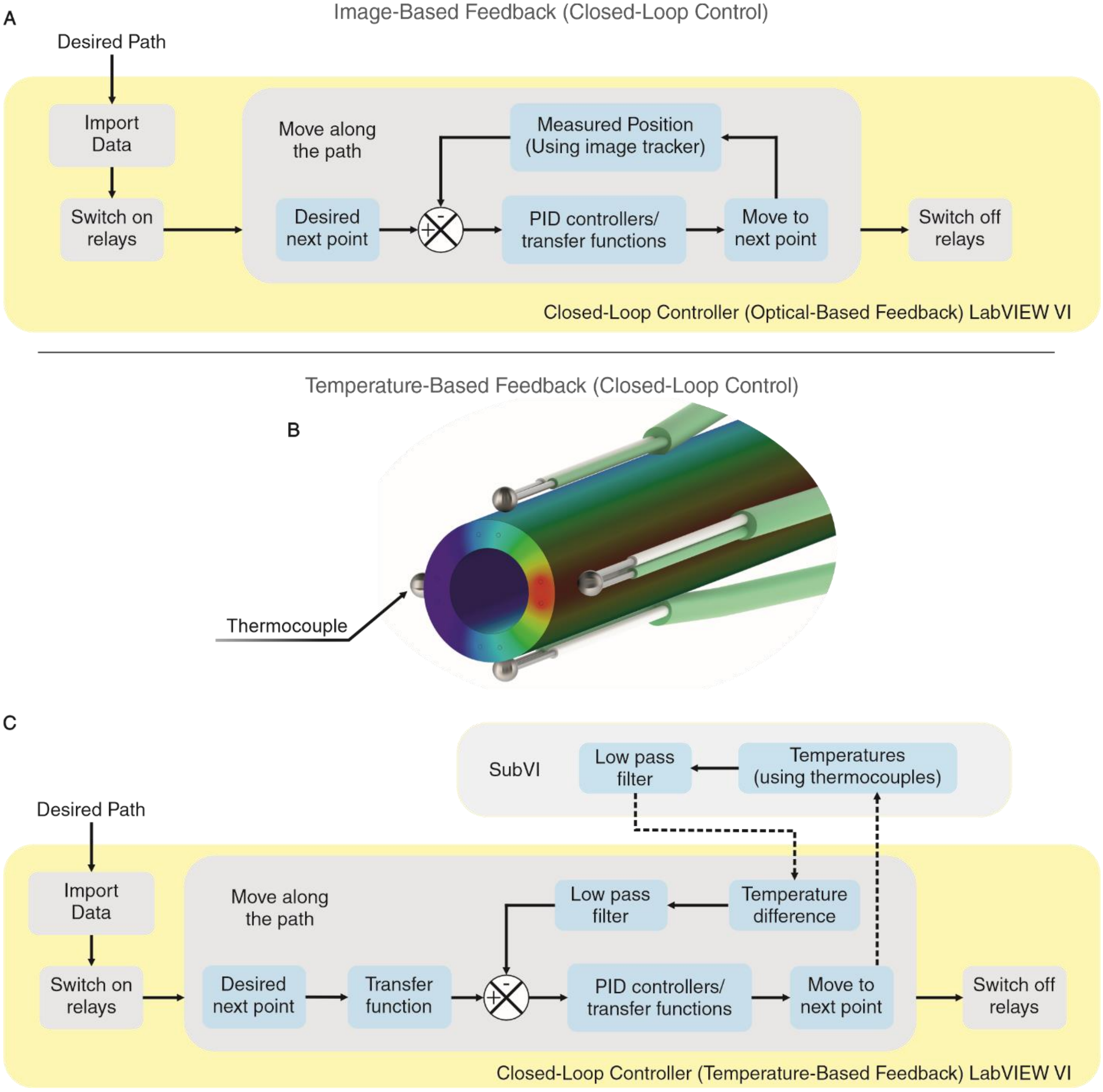

Fig. S10. Closed-loop control mechanisms for fiberbot actuation. (**A**) Flowchart of the control mechanism with microscope image feedback (measured tip's position) used as input to the different PID controllers involved in the automatic estimation of the next trajectory point (target). (**B**) Illustration of four miniature thermocouples attached to the outer surface of the fiber next to the four pairs of wires to provide real-time temperature measurements. (**C**) Flowchart of the control mechanism with temperature signal feedback obtained by the thermocouples placed on opposing sides of the actuated fiber and then fed to the PID controllers (low-pass filtering) to move to the next trajectory position.

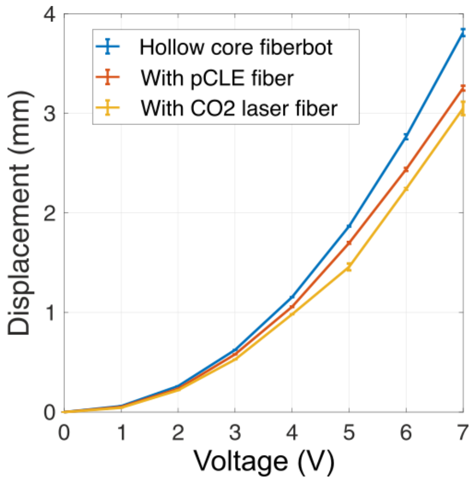

Fig. S11. Characterization of the tip's displacement versus input voltage when the fiberbot is actuated with an instrument (imaging fiber bundle probe – pCLE - and CO_2 laser) inside its central lumen channel.

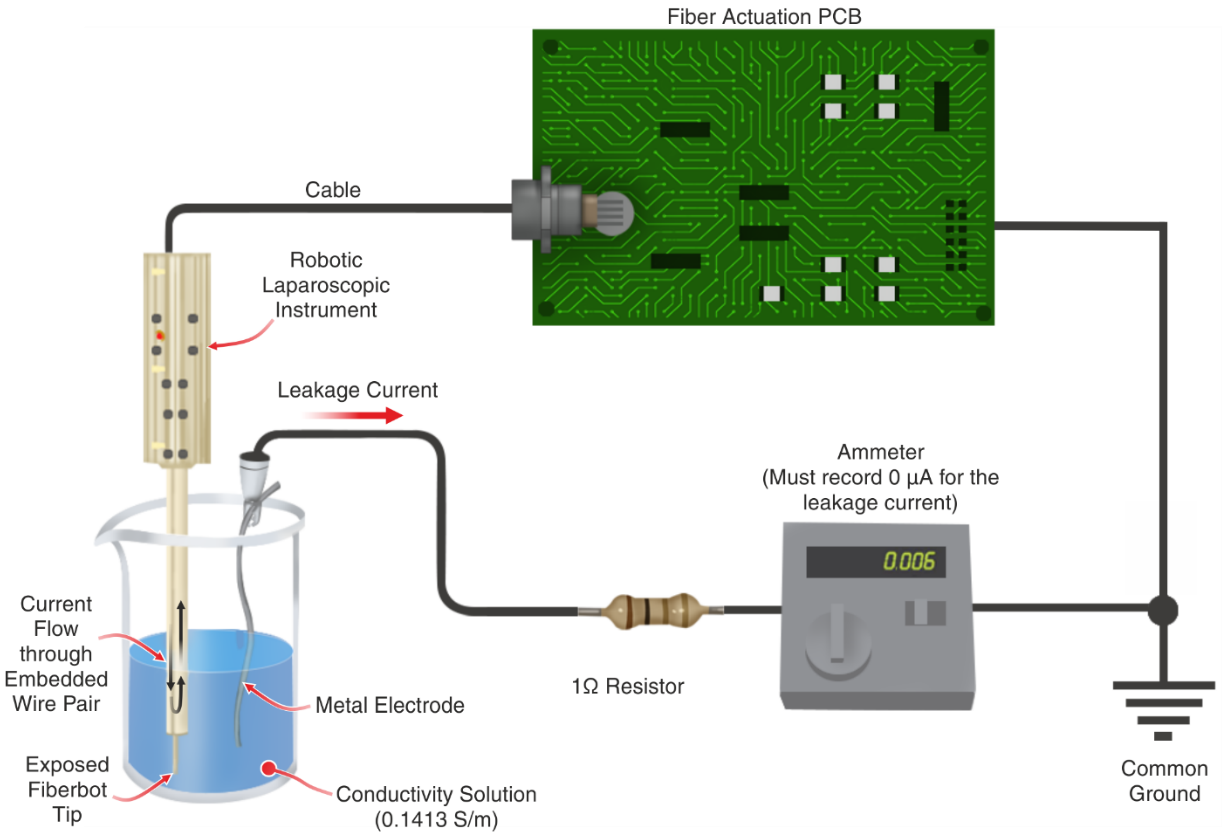

Fig. S12. Setup developed to evaluate the electrical safety of the robotic laparoscopic instrument.

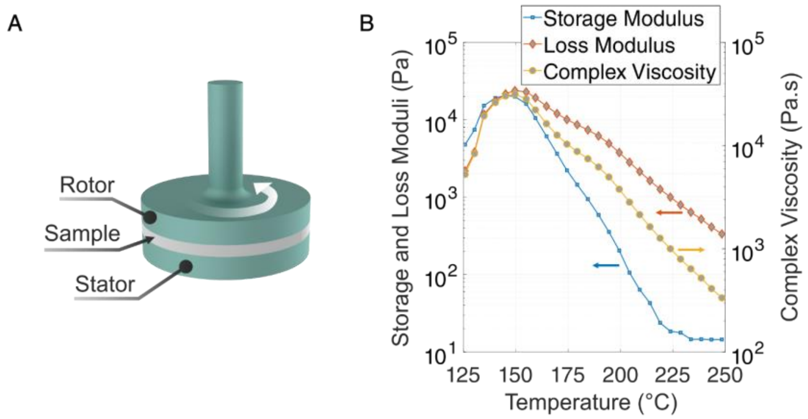

Fig. S13. Rheological properties of 3D printed polycarbonate during a temperature ramp in oscillatory shear rheology. (**A**) Schematic of the testing setup for measuring the storage modulus, loss modulus and complex viscosity of the 3D printed sample. (**B**) The complex viscosity decreases as a function of temperature. The loss modulus decreases and crosses over the decreasing storage modulus. The experiment was conducted using a rheometer with an environmental test chamber (AR2000ex, TA Instruments, USA) at an angular frequency of 1 rad/s and frequency of 0.159 Hz.

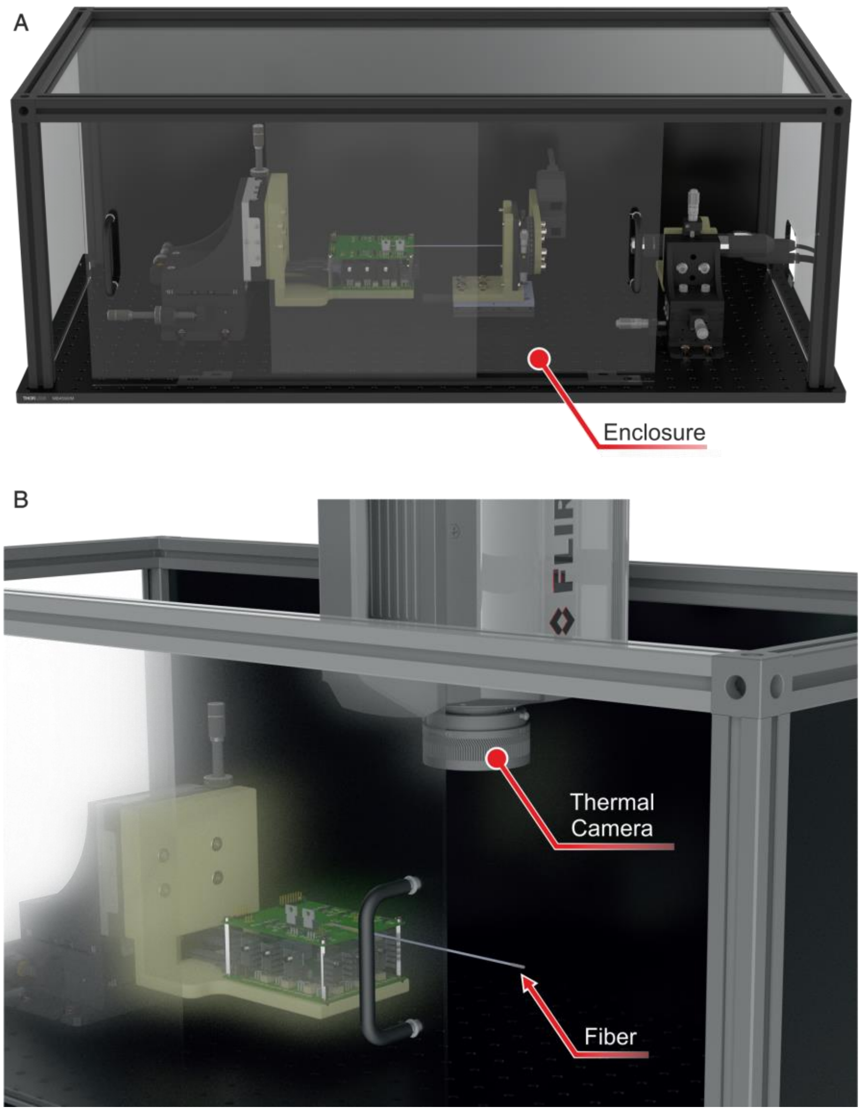

Fig. S14. Setup developed for complete fiberbot motion characterization and coupling to other measurement systems. (**A**) Enclosed case assembled to protect the electrothermal fiber from external environmental sources of light and airflow. (**B**) Positioning of the thermal camera above the fiber setup for free-range movement estimation with thermographic images.

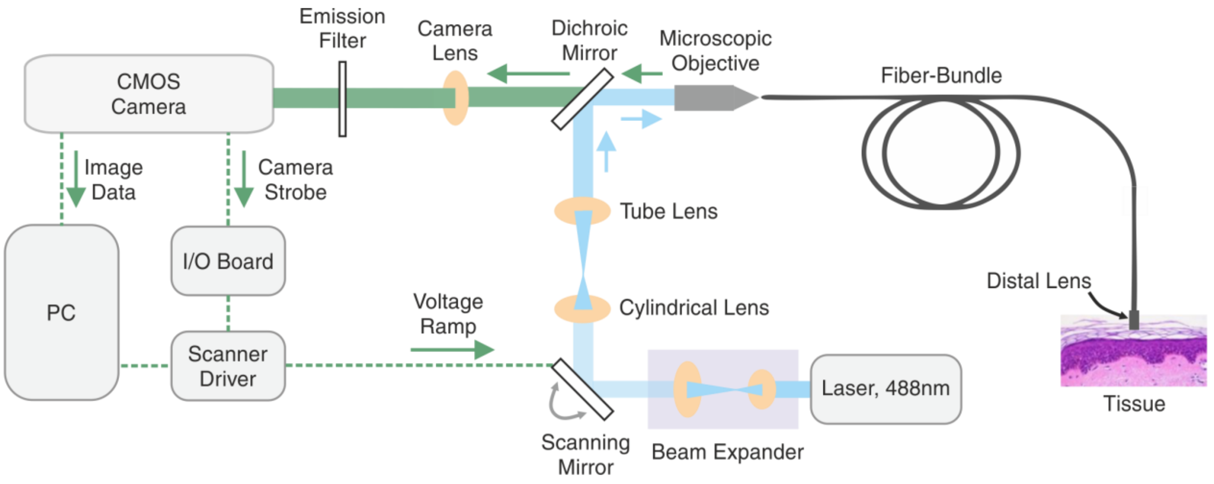

Fig. S15. Line-scan confocal laser endomicroscopy (LS-CLE) system's schematic with main composing modules for light generation, splitting and detection coupled to a fiber-bundle probe for urothelial cancer imaging.

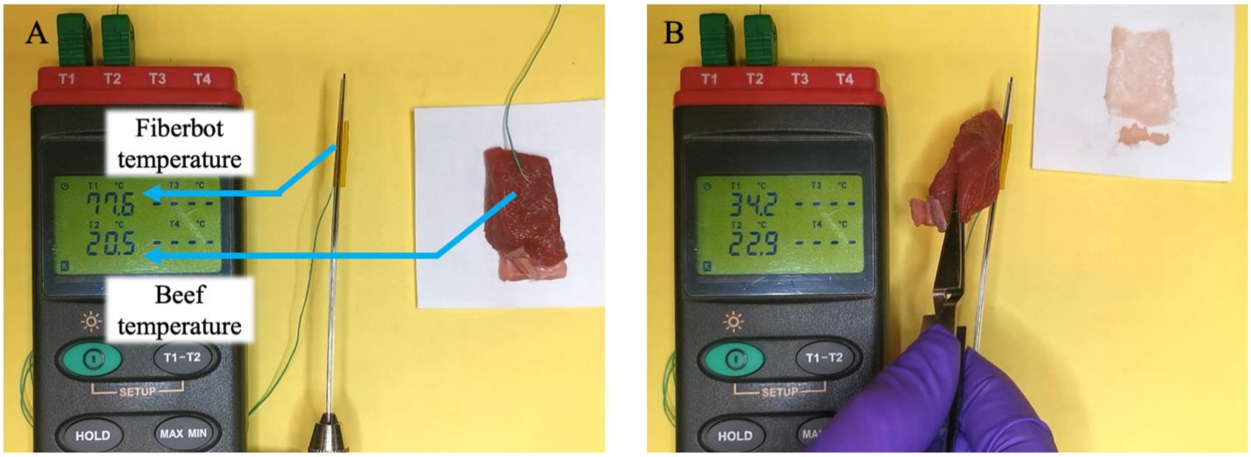

Fig. S16. Temperature testing of fiberbot's outer surface. (**A**) The initial temperature of the outer surface of the actuated fiberbot and beef steak (physical separation) using thermocouples. (**B**) The temperature of the actuated fiberbot when attached to the beef steak.

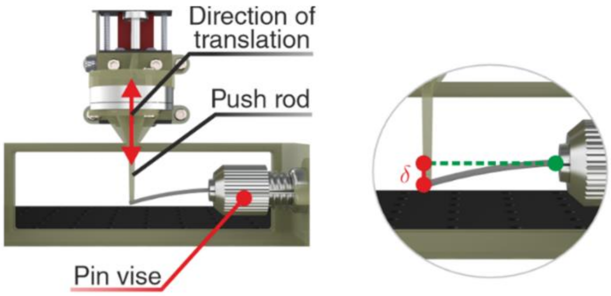

Fig. S17. Characterization setup involved in the determination of the flexural rigidity of the developed fiberbot (polymeric material).

Tables

Table S1. List of various of actuation mechanism

Actuation Method	Wire-driven		CTR	Magnetic		Hydraulic	Piezoelectric/ Electrostrictive	Electrothermal (Our Work)
**Diameter (mm)**	11.5	22	0.09 – 0.43	13	0.4	0.9	(0.24 – 0.46) thickness x (0.68 – 1.3) width	1.65
**Steerable length (mm)**	45	22	*	60	24**	15	35****	120
**Range of motion/ Radius of curvature (mm)**	45 x 45	28ϕ	radius of curvature range (5 – 63)	4 x4	24 x 24	radius of curvature (2.5)	0.08****	5 x 5
**Maximum Speed (mm/s)**	3.5	3	-	94	-	-	*****	10.3
**Reported Errors/ Accuracy [mm]**	RMS error < 0.75	RMS error 0.054 ± 0.028	RMS error 0.201	0.09	100s of Microns***	100s of Microns ***	Submicron***	< 0.05
**References**	*(16)*	*(17)*	*(32)*	*(24)*	*(25)*	*(28)*	*(37)*	-
**Control System**	Motorized actuation module		Motorized actuation module	Large permanent/ Electromagnets		Peristaltic pumps/ Syringe pumps/ Pressure-driven flow controllers	DC high voltage power supply/ High voltage amplifier connected to a function generator	Compact and portable actuation module (Relatively low power)
**Main limitation**	Hysteresis/ Mechanical non-linearities		Sophisticated Control	Large and highly constrained systems		Sophisticated Control/Non-linear behavior of soft materials	High voltage/Small Range of Motion	Thermal effect /Actuator Length

* Variable length, adapting in real-time to the CTR's operational environment due to the flexible interplay of its multiple sliding and rotating tubes.
** Approximation based on the average diameter of the human eyeball.
*** Approximations as exact values were not reported in the publication.
**** The exact dimensions of the fiber used in this experiment were not specified.
***** Instead of illustrating maximum speed in tracing a trajectory, the authors highlighted resonance, reaching a peak second harmonic frequency of approximately 158.3 Hz.

Table S2. Comparison table between performance metrics obtained by different actuation methods for soft robots/actuators published in the literature and the present work.

Ref. & Year	Actuation Method	Device	Dimensions (l = length, w = width)	Maximum Deflection, δl & Strain, δl/l	Electric Power, W (v = voltage, i = current)	Electro-mechanical Factor, δl/l/W	Auxiliary Equipment
(*36*) 2017	Electrostrictive	Microelectromechanical fibre	l = 2.5 cm<br>-	**δl =** 80 μm<br>**δl/l** = 0.0032	-<br>(v = 200 V)	-	External DC high-voltage power source
(24) 2019	Magnetic	Endoscopic laser scan	l = 60 mm<br>w = 13 mm	**δl =** 1.2 mm<br>**δl/l** = 0.02	1.49 W<br>(v = 9 V,<br>i = 0.165 A)	0.0134	Custom-made current control system
(*20*) 2021	Magnetic	Submillimeter-scale soft continuous robot	l = 25 cm<br>w = 600 μm	**δl =** 12 mm<br>**δl/l** = 0.048	30 W<br>(v = 20 V,<br>i = 1.5 A)	0.0016	Helmholtz coil system from MicroMagnetics (Output max: ± 30 V, 2.5 A)
(*25*) 2021	Magnetic	Microcannula for subretinal injection	l = 10 cm<br>w = 400 μm	**δl =** 20 mm<br>**δl/l** = 0.2	336 W[3]<br>(v = 21 V,<br>i = 16 A)	0.0006	OctoMag system (output max: 6 kW, 20 A)
(*84*) 2020	Thermomagnetic	Untethered soft robotic gripper	l = 120 mm<br>w = 15 mm	**δl =** 65 mm<br>**δl/l** = 0.542	50 W	0.0108	Custom-made induction heating system
(*38*) 2018	Piezoelectric	Fibre-bundle probe for endomicroscopy	l = 45 mm<br>w = 5 mm	**δl =** 10 μm<br>**δl/l** = 0.0002	-<br>(v = 100 V)	-	PZT amplifier from PI (output max: ± 250 V, ± 0.1 A)
(*36*) 2021	Piezoelectric	Microrobotic laser steering device	l = 16 mm<br>w = 6 mm	**δl =** 200 μm<br>**δl/l** = 0.0125	-<br>(v = 200 V)	-	PZT amplifier from Advanced Energy Industry Inc. (output max: ± 350 V, ± 0.2 A)
(*44*) 2017	Thermoelectric	Artificial muscle from textiles	l = 21.5 cm<br>w = 0.7 mm	**δl =** 4 mm<br>**δl/l** = 0.0186	2 W<br>(v = 4.5 V,<br>i = 0.45 A)	0.0093	Custom-made half-bridge rectifier
This work 2023	Thermoelectric	Robotic fibre for minimally invasive surgery	l = 12 cm<br>w = 1.65 mm	**δl =** 3.5 mm<br>**δl/l** = 0.0292	1.2 W<br>(v = 12 V,<br>i = 0.1 A)	0.0243	Custom-made voltage-to-current conversion system

Table S3. List of Finite Element Analysis results, illustrating the impact of varying wall thicknesses on the performance of the fiberbot.

Inner Diameter (mm)	Outer Diameter (mm)	Wall Thickness (mm)	Surface Temperature Difference (°C)	Maximum Temperature (°C)	Minimum Temperature (°C)	Tip Displacement (mm)
1.0	1.5	0.25	26.634	78.930	50.283	3.9128
1.0	1.6	0.30	24.238	75.571	49.005	3.7293
1.0	1.7	0.35	22.340	72.787	47.772	3.5404
1.0	1.8	0.40	20.789	70.395	46.594	3.3531
1.0	1.9	0.45	19.491	68.314	45.474	3.1721
1.0	2.0	0.50	18.391	66.475	44.413	2.9996

Table S4. List of Finite element analysis results, illustrating the impact of varying wire positions on the performance of the fiberbot.

Inner Diameter (mm)	Outer Diameter (mm)	Wire Position from Centre "Owp" (mm)	Surface Temperature Difference (°C)	Maximum Temperature (°C)	Minimum Temperature (°C)	Tip Displacement (mm)
1.0	2.0	0.60	15.527	66.475	44.811	3.1585
1.0	2.0	0.65	16.332	66.282	44.684	3.1141
1.0	2.0	0.70	17.285	66.292	44.550	3.0614
1.0	2.0	0.75	18.391	66.475	44.413	2.9996
1.0	2.0	0.80	19.659	66.829	44.272	2.9290
1.0	2.0	0.85	21.075	67.398	44.130	2.8507
1.0	2.0	0.90	22.559	68.250	43.984	2.7760

Table S5. List of Finite element analysis results, illustrating the impact of different fiber materials on the displacement of the fiberbot.

Polymer*	Young's Modulus (10^9 Pa)	CTE (10^{-6} K^{-1})	Thermal Conductivity (W m^{-1} K^{-1})	Poisson's Ratio	Specific Heat (J Kg^{-1} K^{-1})	Tip Displacement (mm)	Ref
PC	2.20 - 2.50	70 – 90	0.19 – 0.22	0.39	1100	3.66	(*75*)
ABS	1.79 – 3.20	80 -100	0.14 – 0.21	0.35 – 0.39	1400 – 1900	4.58	(*85*)
COC	2.60 – 3.20	60 – 70	0.12 – 0.15	0.37 – 0.38	1000	4.23	(*86*)
PMMA	2.50 - 3.50	50 – 90	0.17 – 0.19	0.34 – 0.41	1270	3.81	(*87*)
PETG	1.90 - 2.00	80	0.16 – 0.23	0.40	1100 – 1300	3.56	(*88*)

*PC: Polycarbonate, ABS: Acrylonitrile Butadiene Styrene, COC: Cyclic Olefin Copolymer, PMMA: Poly (methyl methacrylate), PETG: Polyethylene Terephthalate Glycol.

Captions for Movies

Movie S1. First part: The unexpected movement of the thermally drawn fiber when it's placed on top of a hotplate due to asymmetric thermal expansion. Second part: The fiber moves along a circular path with three different actuation speeds and circles' radii. The fiberbot is 1.65 mm in outer diameter and 12 cm in length.

Movie S2. Numerical simulations for the temperature and mechanical displacement of the fiber when actuated through a single pair of wires (left side).

Movie S3. Longitudinal temperature distribution of the actuated fiber using a thermal imaging camera. Fiber is 1.65 mm in outer diameter and 12 cm in length.

Movie S4. The fiber moves along a preoptimized spiral path while simultaneously imaging lens tissue and *ex vivo* bladder tissue using a fiber-bundle-based line-scan confocal laser endomicroscopy system. The fiberbot is 1.65 mm in outer diameter and 12 cm in length.

Movie S5. The fiber moves along a preoptimized path of parallel lines while simultaneously ablating *ex vivo* tissue using an embedded CO_2 laser fiber and collecting the generated aerosol for analysis by the REIMS system. The fiberbot is 1.65 mm in outer diameter and 10 cm in length.

Movie S6. *In vivo* demonstration of the laparoscopic robotic instrument in a porcine model. This movie demonstrates fiberbot's simultaneous actuation and ablation (during the safe apnea phases) for spiral and circular paths in the liver, as well as a circular path in the cecum.

Movie S7. Experimental and animated video for the fiber drawing process.

REFERENCES AND NOTES

1. J. -H. Angelsen, A. Horn, G. Eide, A. Viste, Surgery for colorectal liver metastases: The impact of resection margins on recurrence and overall survival. *World J. Surg. Onc.* **12**, 127 (2014).

2. C. Are, M. Gonen, K. Zazzali, R. P. Dematteo, W. R. Jarnagin, Y. Fong, L. H. Blumgart, M. D'Angelica, The impact of margins on outcome after hepatic resection for colorectal metastasis. *Ann. Surg.* **246**, 295–300 (2007).

3. H. Takashima, M. Moriguchi, N. Hayashi, K. Ikeda, K. Ogiso, C. Yokomizo, H. Uejima, T. Itoh, H. Tomioka, S. Mizuno, S. Shimizu, K. Yasui, Y. Itoh, A simple method to avoid bile duct injury during percutaneous radiofrequency ablation therapy for hepatocellular carcinoma. *Case Rep. Oncol.* **13**, 1337–1342 (2020).

4. L. Crocetti, T. de Baere, R. Lencioni, Quality improvement guidelines for radiofrequency ablation of liver tumours. *Cardiovasc. Interv. Radiol.* **33**, 11–17 (2010).

5. B. Temelkuran, S. D. Hart, G. Benoit, J. D. Joannopoulos, Y. Fink, Wavelength-scalable hollow optical fibres with large photonic bandgaps for CO_2 laser transmission. *Nature* **420**, 650–653 (2002).

6. J. A. Harrington, A review of IR transmitting, hollow waveguides. *Fiber Integr. Opt.* **19**, 211–227 (2000).

7. R. W. Ryan, T. Wolf, R. F. Spetzler, S. W. Coons, Y. Fink, M. C. Preul, Application of a flexible CO_2 laser fiber for neurosurgery: Laser-tissue interactions. *J. Neurosurg.* **112**, 434–443 (2010).

8. A. Villard, I. Breuskin, O. Casiraghi, S. Asmandar, C. Laplace-Buille, M. Abbaci, A. M. Plana, Confocal laser endomicroscopy and confocal microscopy for head and neck cancer imaging: Recent updates and future perspectives. *Oral Oncol.* **127**, 105826 (2022).

9. J. Balog, L. Sasi-Szabó, J. Kinross, M. R. Lewis, L. J. Muirhead, K. Veselkov, R. Mirnezami, B. Dezső, L. Damjanovich, A. Darzi, J. K. Nicholson, Z. Takáts, Intraoperative tissue identification using rapid evaporative ionization mass spectrometry. *Sci. Transl. Med.* **5,** 194ra93 (2013).

10. M. B. Sturm, B. P. Joshi, S. Lu, C. Piraka, S. Khondee, B. J. Elmunzer, R. S. Kwon, D. G. Beer, H. D. Appelman, D. K. Turgeon, T. D. Wang, Targeted imaging of esophageal neoplasia with a fluorescently labeled peptide: First-in-human results. *Sci. Transl. Med.* **5,** 184ra61 (2013).

11. A. R. Akram, S. V. Chankeshwara, E. Scholefield, T. Aslam, N. McDonald, A. Megia-Fernandez, A. Marshall, B. Mills, N. Avlonitis, T. H. Craven, A. M. Smyth, D. S. Collie, C. Gray, N. Hirani, A. T. Hill, J. R. Govan, T. Walsh, C. Haslett, M. Bradley, K. Dhaliwal, In situ identification of gram-negative bacteria in human lungs using a topical fluorescent peptide targeting lipid A. *Sci. Transl. Med.* **10,** eaal0033 (2018).

12. H. C. Lee, N. E. Pacheco, L. Fichera, S. Russo, When the end effector is a laser: A review of robotics in laser surgery. *Adv. Intell. Syst.* **4**, 2200130 (2022).

13. C. D'Ettorre, A. Mariani, A. Stilli, F. R. y Baena, P. Valdastri, A. Deguet, P. Kazanzides, R. H. Taylor, G. S. Fischer, S. P. Di Maio, A. Menciassi, D. Stoyanov, Accelerating surgical robotics research: A review of 10 years with the Da Vinci Research Kit. *IEEE Robot. Autom. Mag.* **28**, 56–78 (2021).

14. M. F. Keating, J. Zhang, C. L. Feider, S. Retailleau, R. Reid, A. Antaris, B. Hart, G. Tan, T. E. Milner, K. Miller, L. S. Eberlin, Integrating the masspec pen to the Da Vinci surgical system for *in vivo* tissue analysis during a robotic assisted porcine surgery. *Anal. Chem.* **92**, 11535–11542 (2020).

15. D. H. Lee, Y. H. Kim, J. Collins, A. Kapoor, D. S. Kwon, T. Mansi, Non-linear hysteresis compensation of a tendon-sheath-driven robotic manipulator using motor current. *IEEE Robot. Autom. Lett.* **6**, 1224–1231 (2021).

16. D. Kundrat, R. Graesslin, A. Schoob, D. T. Friedrich, M. O. Scheithauer, T. K. Hoffmann, T. Ortmaier, L. A. Kahrs, P. J. Schuler, Preclinical performance evaluation of a robotic endoscope for non-contact laser surgery. *Ann. Biomed. Eng.* **49**, 585–600 (2021).

17. M. Zhao, T. J. C. O. Vrielink, A. A. Kogkas, M. S. Runciman, D. S. Elson, G. P. Mylonas, LaryngoTORS: A novel cable-driven parallel robotic system for transoral laser phonosurgery. *IEEE Robot. Autom. Lett.* **5**, 1516–1523 (2020).

18. J. Lussi, S. Gervasoni, M. Mattille, R. Dreyfus, Q. Boehler, M. Reinehr, N. Ochsenbein, B. J. Nelson, U. Moehrlen, Magnetically guided laser surgery for the treatment of twin-to-twin transfusion syndrome. *Adv. Intell. Syst.* **4**, 2200182 (2022).

19. G. Pittiglio, P. Lloyd, T. da Veiga, O. Onaizah, C. Pompili, J. H. Chandler, P. Valdastri, Patient-specific magnetic catheters for atraumatic autonomous endoscopy. *Soft Robot.* **9**, 1120–1133 (2022).

20. J. Lussi, M. Mattmann, S. Sevim, F. Grigis, C. De Marco, C. Chautems, S. Pané, J. Puigmartí-Luis, Q. Boehler, B. J. Nelson, A submillimeter continuous variable stiffness catheter for compliance control. *Adv. Sci.* **8,** e2101290 (2021).

21. Y. Kim, G. A. Parada, S. Liu, X. Zhao, Ferromagnetic soft continuum robots. *Sci. Robot.* **4,** eaax7329 (2019).

22. Y. Kim, E. Genevriere, P. Harker, J. Choe, M. Balicki, R. W. Regenhardt, J. E. Vranic, A. A. Dmytriw, A. B. Patel, X. Zhao, Telerobotic neurovascular interventions with magnetic manipulation. *Sci. Robot.* **7**, eabg9907 (2022).

23. Z. Yang, H. Yang, Y. Cao, Y. Cui, L. Zhang, Magnetically actuated continuum medical robots: A review. *Adv. Intell. Syst.* **5**, 2200416 (2023).

24. A. Acemoglu, D. Pucci, L. S. Mattos, Design and control of a magnetic laser scanner for endoscopic microsurgeries. *IEEE ASME Trans. Mechatron.* **24**, 527–537 (2019).

25. S. L. Charreyron, Q. Boehler, A. N. Danun, A. Mesot, M. Becker, B. J. Nelson, A magnetically navigated microcannula for subretinal injections. *I.E.E.E. Trans. Biomed. Eng.* **68**, 119–129 (2020).

26. B. Gorissen, W. Vincentie, F. Al-Bender, D. Reynaerts, M. De Volder, Modeling and bonding-free fabrication of flexible fluidic microactuators with a bending motion. *J. Micromech. Microeng.* **23**, 045012 (2013).

27. J. Paek, I. Cho, J. Kim, Microrobotic tentacles with spiral bending capability based on shape-engineered elastomeric microtubes. *Sci. Rep.* **5**, 10768 (2015).

28. T. Gopesh, J. H. Wen, D. Santiago-Dieppa, B. Yan, J. S. Pannell, A. Khalessi, A. Norbash, J. Friend, Soft robotic steerable microcatheter for the endovascular treatment of cerebral disorders. *Sci. Robot.* **6**, eabf0601 (2021).

29. I. S. Godage, A. A. Remirez, R. Wirz, K. D. Weaver, J. Burgner-Kahrs, R. J. Webster, Robotic intracerebral hemorrhage evacuation: An in-scanner approach with concentric tube robots, in the *Proceedings of 2015 IEEE/RSJ International Conference on Intelligent Robots and Systems (IROS)* (IEEE, 2015).

30. D. T. Friedrich, V. Modes, T. K. Hoffmann, J. Greve, P. J. Schuler, J. Burgner-Kahrs, Teleoperated tubular continuum robots for transoral surgery - feasibility in a porcine larynx model. *Int. J. Med. Robot.* **14**, e1928 (2018).

31. C. M. Graves, A. Slocum, R. Gupta, C. J. Walsh, Towards a compact robotically steerable thermal ablation probe, in *Proceedings of the 2012 IEEE International Conference on Robotics and Automation* (IEEE, 2012).

32. C. J. Nwafor, G. J. Laurent, P. Rougeot, K. Rabenorosoa, The Caturo: A submillimeter diameter glass concentric tube robot with high curvature, *Adv. Intell. Syst.* **5**, 2200308 (2023).

33. H. Alfalahi, F. Renda, C. Stefanini, Concentric tube robots for minimally invasive surgery: Current applications and future opportunities. *IEEE Trans. Med. Robot. Bionics.* **2**, 410–424 (2020).

34. S. A. Bothner, P. A. York, P. C. Song, R. J. Wood, A compact laser-steering end-effector for transoral robotic surgery, in *Proceedings of the 2019 IEEE/RSJ International Conference on Intelligent Robots and Systems (IROS)* (IEEE, 2019), pp. 7091–7096.

35. S. Patel, M. Rajadhyaksha, S. Kirov, Y. Li, R. Toledo-Crow, Endoscopic laser scalpel for head and neck cancer surgery. *Proc. SPIE* **8207**, 82071S (2012).

36. P. A. York, R. Peña, D. Kent, R. J. Wood, Microrobotic laser steering for minimally invasive surgery. *Sci. Robot.* **6**, eabd5476 (2021).

37. T. Khudiyev, J. Clayton, E. Levy, N. Chocat, A. Gumennik, A. M. Stolyarov, J. Joannopoulos, Y. Fink, Electrostrictive microelectromechanical fibres and textiles. *Nat. Commun.* **8**, 1435 (2017).

38. K. Vyas, M. Hughes, B. G. Rosa, G.-Z. Yang, Fiber bundle shifting endomicroscopy for high-resolution imaging. *Biomed. Opt. Express* **9**, 4649–4664 (2018).

39. Y. Mizutani, Y. Otani, N. Umeda, Micromanipulators comprising optical fiber cantilevers. *Int. J. Optomechatronics* **3**, 18–29 (2009).

40. Y. Zhu, H. D. Espinosa, An electromechanical material testing system for in situ electron microscopy and applications. *Proc. Natl. Acad. Sci. U.S.A* **102**, 14503–14508 (2005).

41. A. Potekhina, C. Wang, Review of electrothermal actuators and applications. *Actuators* **8**, 69 (2019).

42. M. Amjadi, M. Sitti, High-performance multiresponsive paper actuators. *ACS Nano* **10**, 10202–10210 (2016).

43. S. I. Rich, R. J. Wood, C. Majidi, Untethered soft robotics. *Nat. Electron.* **1**, 102–112 (2018).

44. S. M. Mirvakili, I. W. Hunter, Multidirectional artificial muscles from nylon. *Adv. Mater.* **29**, 1604734 (2017).

45. M. Kanik, S. Orguc, G. Varnavides, J. Kim, T. Benavides, D. Gonzalez, T. Akintilo, C. C. Tasan, A. P. Chandrakasan, Y. Fink, P. Anikeva, Strain-programmable fiber-based artificial muscle. *Science* **365**, 145–150 (2019).

46. M. Li, Y. Tang, R. H. Soon, B. Dong, W. Hu, M. Sitti, Miniature coiled artificial muscle for wireless soft medical devices. *Sci. Adv.* **8**, eabm5616 (2022).

47. G.-Z. Yang, J. Bellingham, P. E. Dupont, P. Fischer, L. Floridi, R. Full, N. Jacobstein, V. Kumar, M. Mcnutt, R. Merrifield, B. J. Nelson, B. Scassellati, M. Taddeo, R. Taylor, M. Veloso, Z. L. Wang, R. Wood, The grand challenges of Science Robotics. *Sci. Robot.* **3**, eaar7650 (2018).

48. A. Leber, C. Dong, S. Laperrousaz, H. Banerjee, M. E. M. K. Abdelaziz, N. Bartolomei, B. Schyrr, B. Temelkuran, F. Sorin, Highly integrated multi-material fibers for soft robotics. *Adv. Sci.* **10**, e2204016 (2023).

49. M.-J. Antonini, A. Sahasrabudhe, A. Tabet, M. Schwalm, D. Rosenfeld, I. Garwood, J. Park, G. Loke, T. Khudiyev, M. Kanik, N. Corbin, A. Canales, A. Jasanoff, Y. Fink, P. Anikeeva, Customizing MRI-compatible multifunctional neural interfaces through fiber drawing. *Adv. Funct. Mater.* **31**, 2104857 (2021).

50. A. Perperidis, K. Dhaliwal, S. McLaughlin, T. Vercauteren, Image computing for fibre-bundle endomicroscopy: A review. *Med. Image Anal.* **62**, 101620 (2020).

51. S. E. Mason, E. Manoli, J. L. Alexander, L. Poynter, L. Ford, P. Paizs, A. Adebesin, J. S. McKenzie, F. Rosini, R. Goldin, A. Darzi, Z. Takats, J. M. Kinross, Lipidomic profiling of colorectal lesions for real-time tissue recognition and risk-stratification using rapid evaporative ionization mass spectrometry. *Ann. Surg.* **277**, e569–e577 (2023).

52. M. Tzafetas, A. Mitra, M. Paraskevaidi, Z. Bodai, I. Kalliala, S. Bowden, K. Lathouras, F. Rosini, M. Szasz, A. Savage, E. Manoli, J. Balog, J. McKenzie, D. Lyons, P. Bennett, D. MacIntyre, S. Ghaem-Maghami, Z. Takats, M. Kyrgiou, The intelligent knife (iKnife) and its intraoperative diagnostic advantage for the treatment of cervical disease. *Proc. Natl. Acad. Sci. U.S.A* **117**, 7338–7346 (2020).

53. D. Marcus, D. L. Phelps, A. Savage, J. Balog, H. Kudo, R. Dina, Z. Bodai, F. Rosini, J. Ip, A. Amgheib, J. Abda, E. Manoli, J. McKenzie, J. Yazbek, Z. Takats, S. Ghaem-Maghami, Point-of-care diagnosis of endometrial cancer using the surgical intelligent knife (iKnife)—a prospective pilot study of diagnostic accuracy. *Cancer* **14**, 5892 (2022).

54. M. Paraskevaidi, S. J. S. Cameron, E. Whelan, S. Bowden, M. Tzafetas, A. Mitra, A. Semertzidou, A. Athanasiou, P. R. Bennett, D. A. MacIntyre, Z. Takats, M. Kyrgiou , Laser-assisted rapid evaporative ionisation mass spectrometry (LA-REIMS) as a metabolomics platform in cervical cancer screening. *EBioMedicine* **60**, 103017 (2020).

55. S. J. S. Cameron, Z. Bodai, B. Temelkuran, A. Perdones-Montero, F. Bolt, A. Burke, K. Alexander-Hardiman, M. Salzet, I. Fournier, M. Rebec, Z. Takáts, Utilisation of ambient laser desorption ionisation mass spectrometry (ALDI-MS) improves lipid-based microbial species level identification. *Sci. Rep.* **9**, 3006 (2019).

56. K. M. Langen, D. T. L. Jones, Organ motion and its management. *Int. J. Radiat. Oncol. Biol. Phys.* **50**, 265–278 (2001).

57. M. W. Cornwall, T. G. McPoil, Three-dimensional movement of the foot during the stance phase of walking. *J. Am. Podiatr. Med. Assoc.***89**, 56–66 (1999).

58. S. Fahmi, F. F. J. Simonis, M. Abayazid, Respiratory motion estimation of the liver with abdominal motion as a surrogate. *Int. J. Med. Robot.* **14**, e1940 (2018).

59. J. R. McClelland, D. J. Hawkes, T. Schaeffter, A. P. King, Respiratory motion models: A review. *Med. Image Anal.* **17**, 19–42 (2013).

60. J. Gangloff, R. Ginhoux, M. de Mathelin, L. Soler, J. Marescaux, Model predictive control for compensation of cyclic organ motions in teleoperated laparoscopic surgery. *IEEE Trans. Control Syst. Technol.* **14**, 235–246 (2006).

61. L. Cheng, M. Tavakoli, COVID-19 pandemic spurs medical telerobotic systems: A survey of applications requiring physiological organ motion compensation. *Front. Robot. AI* **7**:594673 (2020).

62. H. Saeidi, J. D. Opfermann, M. Kam, S. Wei, S. Leonard, M. H. Hsieh, J. U. Kang, A. Krieger, Autonomous robotic laparoscopic surgery for intestinal anastomosis. *Sci. Robot.* **7**, eabj2908 (2022).

63. J. Roth, R. Engenhart-Cabillic, L. Eberhardt, N. Timmesfeld, G. Strassmann, Preoxygenated hyperventilated hypocapnic apnea-induced radiation (PHAIR) in breast cancer patients. *Radiother. Oncol.* **100**, 231–235 (2011).

64. R. J. N. Watson, R. Szarko, C. F. Mackenzie, A. J. Sequeira, G. M. Barnas, Continuous endobronchial insufflation during internal mammary artery harvest. *Anesth. Analg.* **75**, 219–225 (1992).

65. L. Machan, L. Churilov, R. Hu, P. Peyton, C. Tan, P. Pillai, L. Ellard, I. Harley, D. Story, P. Hayward, G. Matalanis, N. Roubos, S. Seevanayagam, L. Weinberg, Apneic oxygenation versus low-tidal-volume ventilation in anesthetized cardiac surgical patients: A prospective, single- center, randomized controlled trial. *J. Cardiothorac. Vasc. Anesth.* **31**, 2000–2009 (2017).

66. J. L. Benumof, R. Dagg, R. Benumof, Critical hemoglobin desaturation will occur before return to an unparalyzed state following 1 mg/kg intravenous succinylcholine. *Anesthesiology* **87**, 979–982 (1997).

67. C. Lyons, M. Callaghan, Uses and mechanisms of apnoeic oxygenation: A narrative review. *Anaesthesia* **74**, 497–507 (2019).

68. M. Selvaraj, K. Takahata, Electrothermally driven hydrogel-on-flex-circuit actuator for smart steerable catheters. *Micromachines* **11**, 68 (2020).

69. H. Qiguang, W. Zhijian, W. Yang, M. Adriane, T. Tolley Michael, C. Shengqiang, Electrically controlled liquid crystal elastomer–based soft tubular actuator with multimodal actuation. *Sci. Adv.* **5**, eaax5746 (2019).

70. H. Shirin, B. Shpak, J. Epshtein, J. G. Karstensen, A. Hoffman, R. de Ridder, P. A. Testoni, S. Ishaq, D. N. Reddy, S. A. Gross, H. Neumann, M. Goetz, D. Abramowich, M. Moshkowitz, M. Mizrahi, P. Vilmann, J. W. Rey, S. Sanduleanu-Dascalescu, E. Viale, H. Chaudhari, M. B. Pochapin, M. Yair, M. Shnell, S. Yaari, J. W. Hendel, D. Teubner, R. M. M. Bogie, C. Notaristefano, R. Simantov, N. Gluck, E. Israeli, T. Stigaard, S. Matalon, A. Vilkin, A. Benson, S. Sloth, A. Maliar, A. Waizbard, H. Jacob, P.

Thielsen, E. Shachar, S. Rochberger, T. Hershcovici, J. I. Plougmann, M. Braverman, E. Tsvang, A. A. Abedi, Y. Brachman, P. D. Siersema, R. Kiesslich, G-EYE colonoscopy is superior to standard colonoscopy for increasing adenoma detection rate: An international randomized controlled trial (with videos). *Gastrointest. Endosc.* **89**, 545–553 (2019).

71. M. Alam, D. Ratner, Cutaneous squamous-cell carcinoma. *N. Engl. J. Med.* **344**, 975–983 (2001).

72. R. G. Blanco, P. K. Ha, J. A. Califano, J. M. Saunders, Transoral robotic surgery of the vocal cord. *J. Laparoendoc. Adv. Surg. Tech. A* **21**, 157–159 (2011).

73. M. Arbyn, C. W. Redman, F. Verdoodt, M. Kyrgiou, M. Tzafetas, S. Ghaem-Maghami , K.-U. Petry, S. Leeson, C. Bergeron, P. Nieminen, J. Gondry, O. Reich, E. L. Moss, Incomplete excision of cervical precancer as a predictor of treatment failure: A systematic review and meta-analysis. *Lancet Oncol.* **18**, 1665–1679 (2017).

74. M. Keshavarz, D. J. Wales, F. Seichepine, M. E. M. K. Abdelaziz, P. Kassanos, Q. Li, B. Temelkuran, H. Shen, G.-Z. Yang, Induced neural stem cell differentiation on a drawn fiber scaffold–toward peripheral nerve regeneration. *Biomed. Mater.* **15**, 055011 (2020).

75. M. Ghebrebrhan, G. Z. J. Loke, Y. Fink, Fabrication and measurement of 3D printed retroreflective fibers. *Opt. Mater. Express* **9**, 3432–3438 (2019).

76. T. Nguyen-Dang, Tailoring surface properties of fiber materials: Novel opportunities in the fabrication of multi-scale fiber-based architectures, thesis, École Polytechnique FÉdÉrale de Lausanne, Lausanne, Switzerland (2017).

77. G. Dagnino, J. Liu, M. E. M. K. Abdelaziz, W. Chi, C. Riga, G.-Z. Yang, Haptic feedback and dynamic active constraints for robot-assisted endovascular catheterization, in *Proceedings of the 2018 IEEE/RSJ International Conference on Intelligent Robots and Systems (IROS)* (IEEE, 2018), pp. 1770–1775.

78. J. Dhanda, A. Schache, M. Robinson, Z. Bodai, E. White, B. Temelkuran, G.-Z. Yang, Z. iKnife Rapid evaporative ionisation mass spectrometry (REIMS) technology in head and neck surgery. A ex vivo feasibility study. *Br. J. Oral Maxillofac. Surg.* **55**, e61 (2017).

79. M. Hughes, G.-Z. Yang, Line-scanning fiber bundle endomicroscopy with a virtual detector slit. *Biomed. Opt. Express* **7**, 2257–2268 (2016).

80. T. Vercauteren, A. Meining, F. Lacombe, A. Perchant, Real time autonomous video image registration for endomicroscopy: Fighting the compromises, in *Three-Dimensional and Multidimensional Microscopy: Image Acquisition and Processing XV* (SPIE BiOS, 2008) vol. 68610, pp. 90–97.

81. G. Grimnes, O. G. Martinsen, *Bioimpedance and Bioelectronics Basics* (Academic Press, ed. 2, 2008).

82. C. Gabriel, A. Peyman, E. H. Grant, Electrical conductivity of tissue at frequencies below 1 MHz. *Phys. Med. Biol.* **54**, 4863–4878 (2009).

83. E. Hernández-Balaguera, E. López-Dolado, J. L. Polo, Obtaining electrical equivalent circuits of biological tissues using the current interruption method, circuit theory and fractional calculus. *RCS Adv.* **6**, 22312–22319 (2016).

84. S. M. Mirvakili, D. Sim, I. W. Hunter, R. Langer, Actuation of untethered pneumatic artificial muscles and soft robots using magnetically induced liquid-to-gas phase transitions. *Sci. Robot.* **5**, eaaz4239, 2020.

85. T. H. R. Marques, B. M. Lima, J. H. Osorio, L. E. da Silva, C. M. B. Cordeiro, 3D printed microstructured optical fibers, in *Proceedings of the 2017 SBMO/IEEE MTT-S International Microwave and Optoelectronics Conference (IMOC)* (IEEE, 2017), pp. 1–3.

86. J. J. Kaufman, C. Bow, F. A. Tan, A. M. Cole, A. F. Abouraddy, 3D printing preforms for fiber drawing and structured functional particle production, in *Photonics and Fiber Technology* (Optica Publishing Group, 2016), p. AW4C.1.

87. M. Zubel, A. Fasano, G. Woyessa, K. Sugden, H. R. Rasmussen, O. Bang, 3D-printed PMMA preform for hollow-core POF drawing, in *Proceedings of the 25th International Conference on Plastic Optical Fibers (POF)* (Aston University, 2016).

88. W. Talataisong, R. Ismaeel, T. H. R. Marques, S. A. Mousavi, M. Beresna, M. A. Gouveia, S. R. Sandoghchi, T. Lee, C. M. B. Cordeiro, G. Brambilla, Mid-IR hollow-core microstructured fiber drawn from a 3D printed PETG preform. *Sci. Rep.* **8**, 8113 (2018).
